# The omega subunit of the RNA polymerase core directs transcription efficiency in cyanobacteria

**DOI:** 10.1093/nar/gku084

**Published:** 2014-01-28

**Authors:** Liisa Gunnelius, Kaisa Hakkila, Juha Kurkela, Hajime Wada, Esa Tyystjärvi, Taina Tyystjärvi

**Affiliations:** ^1^Department of Biochemistry, University of Turku, FIN-20014 Turku, Finland and ^2^Department of Life Sciences, University of Tokyo, Komaba 3-8-1, Meguro-ku, Tokyo 153-8902, Japan

## Abstract

The eubacterial RNA polymerase core, a transcription machinery performing DNA-dependent RNA polymerization, consists of two α subunits and β, β′ and ω subunits. An additional σ subunit is recruited for promoter recognition and transcription initiation. Cyanobacteria, a group of eubacteria characterized by oxygenic photosynthesis, have a unique composition of the RNA polymerase (RNAP) core due to splitting of the β′ subunit to N-terminal γ and C-terminal β′ subunits. The physiological roles of the small ω subunit of RNAP, encoded by the *rpoZ* gene, are not yet completely understood in any bacteria. We found that although ω is non-essential in cyanobacteria, it has a major impact on the overall gene expression pattern. In ΔrpoZ strain, recruitment of the primary σ factor into the RNAP holoenzyme is inefficient, which causes downregulation of highly expressed genes and upregulation of many low-expression genes. Especially, genes encoding proteins of photosynthetic carbon concentrating and carbon fixing complexes were down, and the ΔrpoZ mutant showed low light-saturated photosynthetic activity and accumulated photoprotective carotenoids and α-tocopherol. The results indicate that the ω subunit facilitates the association of the primary σ factor with the RNAP core, thereby allowing efficient transcription of highly expressed genes.

## INTRODUCTION

The eubacterial RNA polymerase (RNAP) core, a transcription machinery performing DNA-dependent RNA polymerization, consists of two α subunits and β, β′ and ω subunits. For the RNAP holoenzyme, capable of transcription initiation, an additional σ subunit is recruited. The function of the small ω subunit of RNAP, encoded by the *rpoZ* gene, is not yet completely understood. A structural model of the *Thermus thermophilus* RNAP holoenzyme shows that the ω subunit contacts with the N and C-terminal parts of the β′ subunit (1). Studies with *Escherichia coli* have shown that the ω subunit binds to full length β′ and assists the final step of RNAP core assembly when β′ associates with an α_2_β sub-complex (2). However, completely segregated *rpoZ* deletion strains of *E. coli* (3), *Streptomyces kasugaensis* (4), *Streptomyces coelicolor* (5) and *Mycobacterium smegmatis* (6) have revealed that ω is not an essential subunit. On the contrary, the RPB6 subunit of eukaryotic RNAP I, II and III, and the archaean RpoK subunit, which show sequence, structure and function homology with the ω subunit, are essential proteins (7).

The *rpoZ* deletion strains in *M. smegmatis* and *S. coelicolor* show altered cell surface properties and colony morphology, and the deletion strain of *S. coelicolor* lost antibiotic production capacity (5,6). In *E. coli,* the slow-growth phenotype of an *rpoZ* inactivation strain was shown to be caused by a polarity effect on the adjacent *spoT* gene encoding a pyrophosphatase that regulates the amounts of the stringent response alarmone molecules ppGpp and pppGpp (3). Recently, lack of the ω subunit was suggested to increase DNA relaxation in *E. coli* (8).

Cyanobacteria are eubacteria characterized by oxygenic photosynthesis. The cyanobacterial RNAP has unique features. First, the β′ subunit is split, the N-terminal part is called γ and the C-terminal part retains the name β′ (9). Second, the β′ subunits harbors a large and >600 amino acid long lineage-specific insertion (10). A similar assembly function of the ω subunit as suggested in the other eubacteria would require simultaneous connection of the ω subunit to both γ and β′ subunits.

In this work, we constructed a ΔrpoZ strain of the cyanobacterium *Synechocystis* sp. PCC 6803 (hereafter *Synechocystis*). The ΔrpoZ strain grew as well as the control strain (CS) in standard conditions and contained a normal amount of RNA, but showed major physiological differences to CS, including a low light-saturated photosynthetic activity and a high carotenoid content. Our results show that the RNAP holoenzyme contains less of the primary σ factor SigA in ΔrpoZ than in CS. We suggest that this explains downregulation of many highly expressed genes and simultaneous upregulation of low-expression genes in ΔrpoZ.

## MATERIALS AND METHODS

### Strains and growth conditions

**Figure 1.**
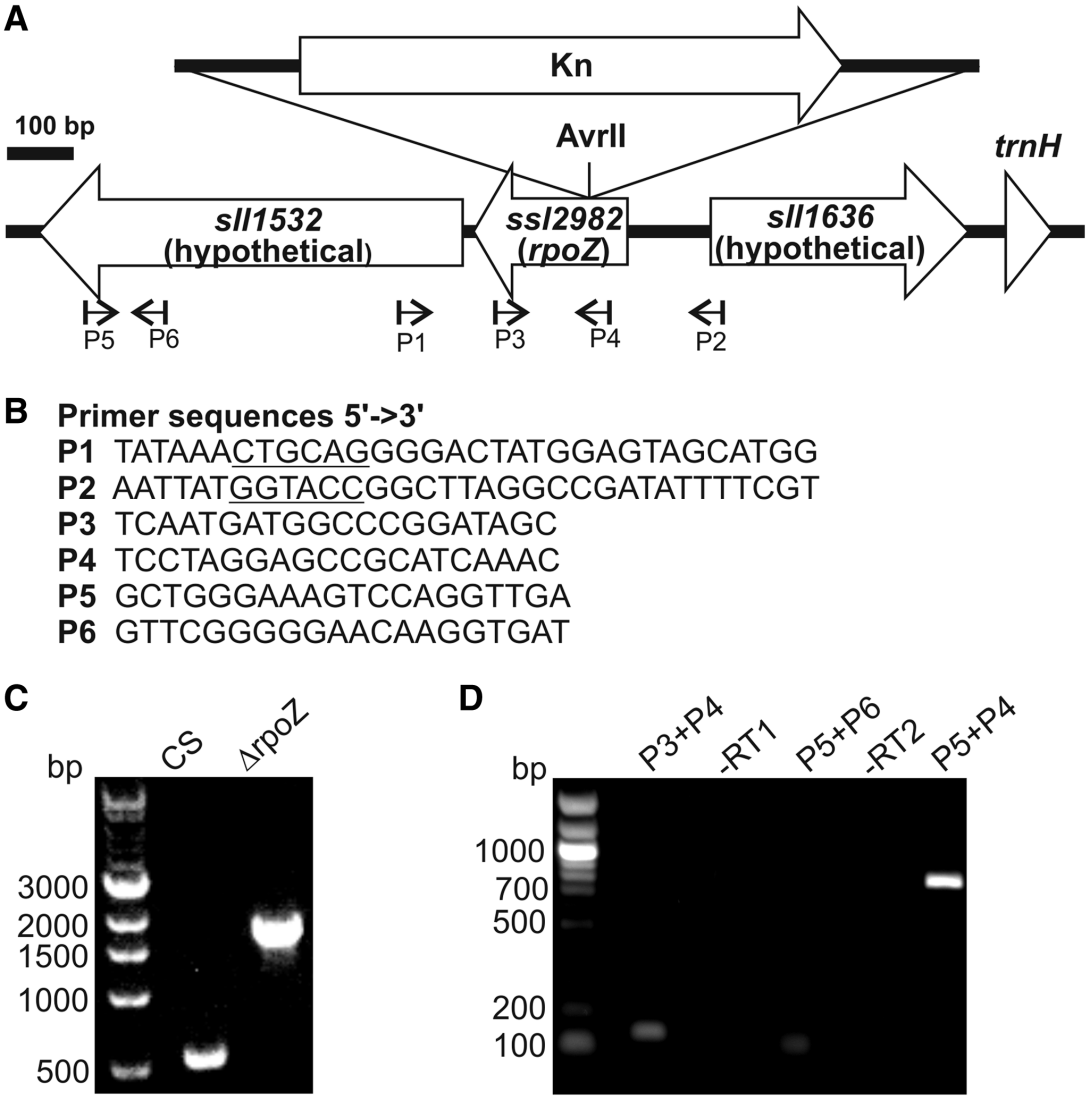
The rpoZ gene region of the genome and construction of the ΔrpoZ strain (**A**) A schematic drawing of the *rpoZ* region of the genome and a ΔrpoZ strain. Primers P1 and P2 were used for construction and PCR verification of the mutant strain, the kanamycin cassette (Kn) was inserted in the middle of the *rpoZ* gene in antisense orientation. Primers P3, P4, P5 and P6 were used in the reverse transcription and subsequent PCR analysis. (**B**) Sequences of primers P1-P6. (**C**) PCR analysis of the ΔrpoZ strain. Genomic DNA was isolated from the control (CS) and ΔrpoZ strains, and the rpoZ gene was amplified. Expected fragment sizes are 533 and 1745 bp for the control and ΔrpoZ strains, respectively. (**D**) RNA was isolated from CS, and reverse transcription was performed using the primer P3 or P5, and then complementary DNA was amplified by PCR. The –RT1 and –RT2 denotes control reactions for primers P3 and P5, respectively, where no reverse transcriptase was added. The predicted PCR product sizes are 130 bp (P3 + P4), 78 bp (P5 + P6) and 795 bp (P5 + P4).

The glucose-tolerant strain of *Synechocystis* sp. PCC 6803 (11) was used as a CS. To construct the ΔrpoZ inactivation strain (Figure 1A), the *rpoZ* gene with flanking sequences was amplified by polymerase chain reaction (PCR) using primers P1 and P2 (Figure 1B) and ligated into KpnI- and PstI-digested pUC19. A fragment containing the kanamycin resistance cassette was amplified from pUC4K and inserted into the AvrII site in the pUC19-rpoZ. CS was transformed with the pUC19-rpoZ:Kn. Transformants were selected on BG-11 agar plates supplemented with kanamycin (50 µg/ml). Complete segregation was confirmed with PCR analysis (Figure 1C).

For the complementation strain, *rpoZ* was amplified with 5′-AATTATGGTACCGGGACTATGGAGTAGCATGG-3′ and 5′-ATATTACATATGATGACCAAGCGTAGTAATTTGG-3′ and ligated into KpnI- and NdeI-digested pAII:Sm (a generous gift from Dr Marion Eisenhut), a plasmid containing the up- and downstream regions of the *psbA2* gene, and this construct was used to transform ΔrpoZ cells to replace the coding region of *psbA2* with the coding region of *rpoZ*. Transformants were selected on BG-11 agar plates supplemented with kanamycin (50 µg/ml), spectinomycin (20 µg/ml) and streptomycin (10 µg/ml).

Cells were grown in standard conditions, in BG-11 medium supplemented with 20 mM Hepes-NaOH, pH 7.5, under continuous illumination at the photosynthetic photon flux density (PPFD) of 40 µmol/(m^2^ s), at 32°C in ambient CO_2_. The BG-11 agar plates for ΔrpoZ were supplemented with kanamycin (50 µg/ml) and those for complementation strain with kanamycin (50 µg/ml), spectinomycin (20 µg/ml) and streptomycin (10 µg/ml). For the experiments, all strains were grown without antibiotics in liquid BG-11 medium for 1–5 days.

### The *rpoZ-sll1532* operon

Reverse transcription-PCR was performed to judge whether of the *rpoZ* and *sll1532* genes belong to the same operon. DNA-free RNA was extracted from CS cells as described for the DNA microarray experiments. Reverse transcription (RT) was performed for 900 ng RNA using SuperScript III kit (Invitrogen) according to the manufacturer’s instructions using the primer P3 for *rpoZ* or the primer P5 for *sll1532* (Figure 1B). For control reactions, no reverse transcriptase was added. Then 2 µl of each RT reaction was used as a template to amplify *rpoZ* (primers P3 and P4), *sll1532* (primers P5 and P6) and possible dicistronic *rpoZ-sll1532* (primers P5 and P4) using Phusion HeatShock II (Thermo Scientific) enzyme (Figure 1B). The following program was used: initial denaturation (30 s at 98°C); 25 cycles of denaturation (10 s at 98°C), annealing (30 s at 51°C) and polymerization (30 s at 72°C); final polymerization (5 min at 72°C). Five microliters of the PCR reaction was loaded on 1% agarose gel and stained with ethidium bromide (Figure 1D).

### Isolation of total RNA and DNA microarray

Total RNA was isolated from 1 ml of cell culture (OD_730_ = 1) as described previously (12) from three independent biological replicates, and RNA concentration was measured; for visualization, RNAs were separated in 1.2% agarose gel and stained with ethidium bromide.

For transcription profiling, CS and ΔrpoZ were grown in standard conditions for 72 h (OD_730_ = 1; 3.5 µg of chlorophyll (Chl) *a*/ml; 20 ml). Total RNA was isolated from three (ΔrpoZ) or four (CS) independent biological replicates with the hot-phenol method and then purified with RNeasy kit (Qiagen). Microarray experiments were performed using an Agilent 8 × 15 K custom cyanobacterium *Synechocystis* sp. PCC 6803 array (13). RNA labeling, data collection and normalization were done as described previously (14). A gene was considered upregulated if log_2_ of the fold change (FC) was ≥1 and downregulated if FC was less than and equal to −1, and the results were statistically significant (*P* < 0.05).

For estimation of gene expression levels in CS, both raw microarray data and data after normalization with the quantile method were analyzed. The signal intensity for each gene was calculated as the mean intensity obtained from all probes representing the gene in the DNA microarray, and the mean of the log_2_ of the signal value in four independent biological replicates was used as the final value. The data shown are after normalization but both methods gave similar results, as the lists of 100 most highly expressed genes were 96% similar.

#### Sequence logos of promoter regions

The 60-nt long 5′ upstream sequences of *Synechocystis* genes for which a transcription start site has been experimentally determined (15) and for which FC less than and equal to −1 or FC more than and equal to +1 in ΔrpoZ compared with CS were aligned. To calculate the −10 region, the sequences were aligned so that the information content (16) of a 6-nt long subsequence between nucleotides −14 and −5 was maximized. The −35 region was then obtained by maximizing the information content of a 6-nt long subsequence placed 16–18 nt upstream from the −10 region. The logos were drawn with Weblogo (http://weblogo.berkeley.edu).

#### Western blotting

Cells (25 ml; OD_730_ = 1; 3.5 µg of Chl *a*/ml) were harvested from standard growth conditions. Total proteins and membrane proteins were isolated as described previously (17). Protein samples containing 1.6 µg (allophycocyanin, phycocyanin), 5 µg (CP43, rubisco), 10 ug (PsaB, NdhJ, NdhK), 20 µg (ω), 40 µg (Flv3) or 50 µg (α and β subunits of RNAP, SigA, HspA, NdhD3, Flv2) total protein were solubilized for 10 min at 75°C and separated by 10% NEXT GEL™ sodium dodecyl sulfate–polyacrylamide gel electrophoresis (SDS–PAGE) (Amresco) according to the manufacturer's instructions. For AtpE, 60 µg of membrane proteins was used instead of total proteins. Proteins were transferred to Immobilon-P membrane (Millipore). Antibodies against allophycocyanin (AS08 277), phycocyanin (AS08 278), PsaB (AS10 695), CP43 (AS11 1787) and HspA (AS08 286) were purchased from Agrisera; antibodies against Flv2, Flv3, NdhJ, NdhK and NdhD3 were gifts from Prof. E-M. Aro. Custom polyclonal antibodies against peptides CKSYTDQPQIGRLTA, CIRVQPHSPDNPAEK, CVAATEGKEKKVRKI and CMSDELTRPEIISDN recognizing α, β, SigA and ω subunits of RNAP, respectively, were purchased from Agrisera. The goat anti-rabbit IgG (H+L) alkaline phosphatase conjugate (Zymed) and the CDP star chemiluminescence kit (New England Biolabs) were used for detection in western blotting. At least three independent biological replicates were analyzed, and immunoblots were quantified with a FluorChem image analyzer (Alpha Innotech Corp.).

#### Free and RNAP bound SigA

Cells (30 ml, OD_730_ = 1) were harvested from the standard growth conditions, and soluble proteins were isolated as described previously (18) without a freezing step, and using buffer 50 mM Tris–HCl, pH 7.6, 0.15 M NaCl, 10 mM EDTA supplemented with protease inhibitor cocktail tablet (Roche). Protein samples containing 250 μg of soluble proteins were filtered through an Amicon Ultra-0.5 ml 100K column (Millipore), and the flow through was further filtered with 30 K column, to obtain fractions containing the RNAP complex and free SigA and α subunits, respectively. Equal amounts of fractions were loaded on gels. Samples were analyzed as in western blotting using antibodies against α and SigA subunits of RNAP.

#### Growth, photosynthetic activity and 77K fluorescence measurements

Growth was measured as described previously (19). Light-saturated photosynthetic activity *in vivo* was measured (1 ml samples, 3.5 µg of Chl *a*/ml) with a Clark-type oxygen electrode (Hansatech Ltd) at 32°C in BG-11 medium supplemented with 10 mM NaHCO_3_. Fluorescence emission spectra were measured at 77 K with an Ocean Optics S2000 spectrometer by exciting the sample (OD_730_ = 5, 50 µl) with light from a slide projector through a 440-nm line filter (Corion, Dunedin FL, USA). The spectra were corrected by subtracting a background at 615 nm, smoothened by a moving median with a 2-nm window and normalized by dividing by the peak value of PSI emission at 723 nm.

#### Pigment, α-tocopherol and lipid contents

*In vivo* absorption spectra were measured with a UV-3000 spectrophotometer (Shimadzu) from 400 to 800 nm. The carotenoid peak at 678 nm, the phycobilin peak at 625 nm and the Chl *a* peak at 678 nm were used for pigment analysis. The Chl *a* content of intact cells was measured as described previously (19). Carotenoids and α-tocopherol were extracted with methanol and detected by high pressure (or high performance) liquid chromatography (HPLC) as described earlier, except that a washing step of cell pellets was omitted (19). Lipids were extracted from intact cells by the method of Blight and Dyer (20). Lipid classes were separated with thin-layer chromatography and quantified by gas chromatography as described previously (21).

## RESULTS AND DISCUSSION

### The ω subunit is non-essential in cyanobacteria

Cyanobacterial ω subunits form a monophyletic clade in a phylogenetic tree containing representatives of the main eubacterial groups (Supplementary Figure S1). The cyanobacterial clade includes chloroplast-encoded ω subunits of green and red algae and cyanelle-encoded ones of glaucocystophytes, but the ω subunit appears to be lost from plastomes of higher plants (Supplementary Figure S1).

Like many other *Synechocystis* sp. PCC 6803 laboratory strains, our glucose tolerant CS does not contain the *slr1635* transposase next to the *rpoZ* (*ssl2982*) gene, unlike the originally sequenced Kazusa strain (Figure 1 and Supplementary Figure S2). The coding regions of *rpoZ* and adjacent *sll1532* are separated only by 31 bp. Our analysis showed that the *rpoZ* gene forms an operon together with *sll1532* that encodes a hypothetical protein (Figure 1D).

The ΔrpoZ mutant was constructed by inserting a kanamycin resistance cassette in antisense orientation to the middle of the *rpoZ* gene (Figure 1A). Complete segregation of the ΔrpoZ mutant strain was verified by PCR (Figure 1C). A complementation strain was constructed to verify that the phenotype of ΔrpoZ was due to inactivation of the *rpoZ* gene. In the complementation strain, the coding region of the *psbA2* gene was replaced by the coding region of the *rpoZ* gene in the ΔrpoZ strain. The phenotype of the complementation strain was similar to that of CS, except that the ω subunit was overexpressed, as it was under the strong *psbA2* promoter (Supplementary Figure S3).

**Figure 2.**
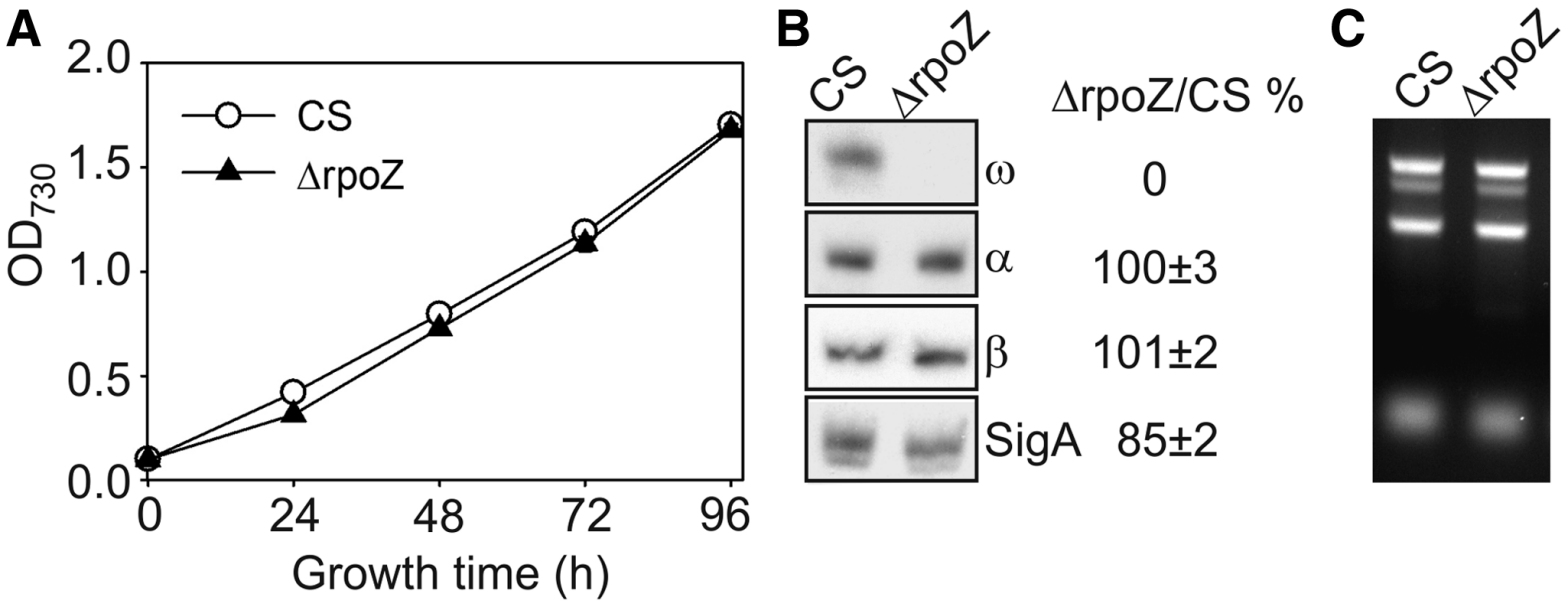
Growth, RNAP and RNA contents of the control (CS) and ΔrpoZ strains. (**A**) Cells were grown in standard conditions under continuous illumination at the PPFD of 40 µmol/(m^2^s), 32°C, air level CO_2_. Each data point represents an average of six independent cultures, and the error bars denote SE (shown if larger than the symbols). (**B**) Total proteins were isolated from cells grown in standard conditions, and α, β, ω and SigA subunits of RNAP were detected by western blotting. Subunit content in ΔrpoZ is expressed as percentage of that measured in CS. (**C**) Total RNA was isolated from 1 ml of cell culture with OD_730_ = 1, and the RNAs were separated with 1.2% agarose gel and stained with ethidium bromide.

The ΔrpoZ strain grew as well as CS in standard growth conditions (Figure 2A). Western blot analysis confirmed the absence of the ω subunit in ΔrpoZ (Figure 2B). Similar amounts of α and β subunits of RNAP were detected in ΔrpoZ and CS, but the amount of the primary σ factor, SigA, was slightly lower in ΔrpoZ than in CS (Figure 2B). The RNA contents of ΔrpoZ and CS were similar, 1 ml of cells culture (OD_730_ = 1) contained 1.28 ± 0.05 and 1.18 ± 0.14 µg RNA in CS and ΔrpoZ, respectively (Figure 2C). This indicates that at least the abundant ribosomal RNA is similarly produced in both strains. Our data also suggest that the RNAP core content remains normal in cyanobacteria lacking the ω subunit, but the lowered SigA content of ΔrpoZ may cause changes in the transcriptional pattern.

### Inactivation of rpoZ reorganizes transcription pattern

**Figure 3.**
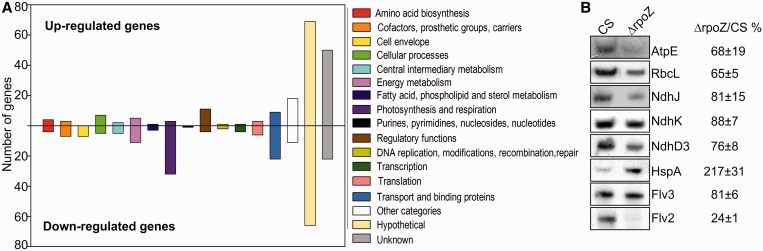
Upregulated and downregulated genes in ΔrpoZ. (**A**) The 187 genes upregulated at least 2-fold, and the 212 genes downregulated to one half or less were arranged to gene categories according to Cyanobase. (**B**) Total proteins were isolated and the amounts of AtpE, RbcL, NdhJ, NdhK, NdhD3, HspA, Flv3 and Flv2 proteins were detected by western blotting.

A DNA microarray analysis was performed in standard conditions. In ΔrpoZ, 187 genes were at least 2-fold upregulated and 212 genes were downregulated to one half or less. The distribution of upregulated and downregulated genes to different gene categories according to Cyanobase (http://genome.microbedb.jp/cyanobase) is shown in Figure 3A and, full lists of upregulated and downregulated genes are shown in Supplementary Tables S1 and S2, respectively. All microarray data are available in GEO (accession GSE51647). Genes for energy metabolism, photosynthesis and transport were largely downregulated in ΔrpoZ, whereas many regulatory genes and genes with unknown functions were upregulated (Figure 3A). The downregulated genes included both the *rpoZ* gene and the adjacent *sll1532*.

**Figure 4.**
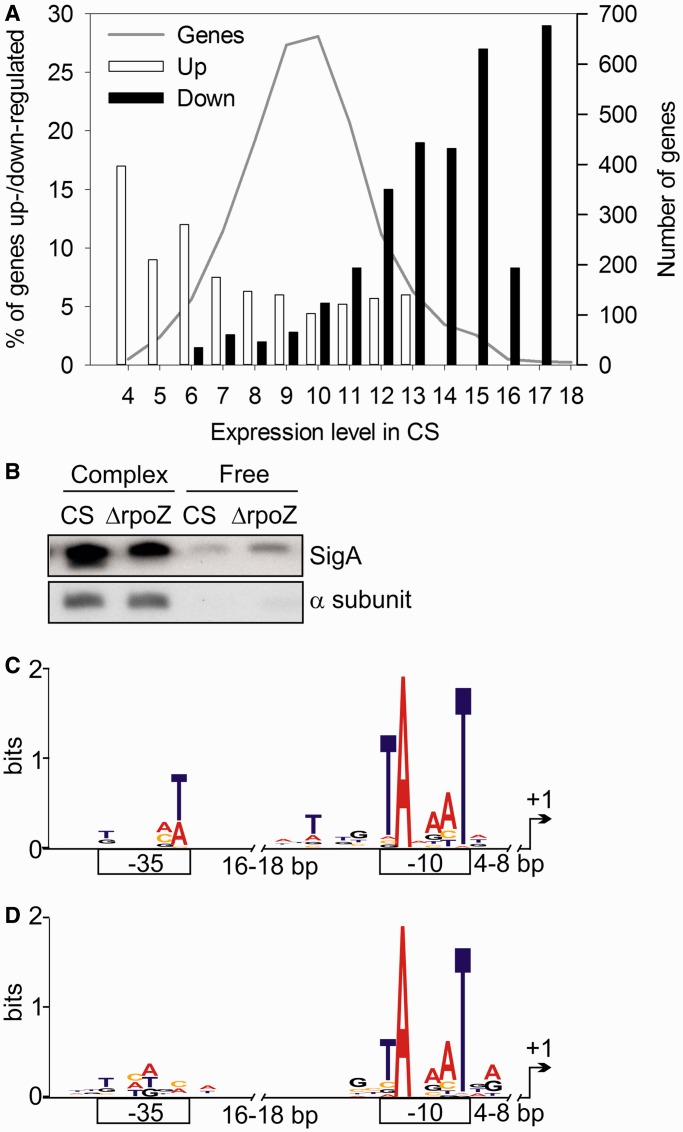
Characteristics of upregulated and downregulated genes in ΔrpoZ. (**A**) Relationship between gene expression level and upregulation or downregulation of the gene in ΔrpoZ. The log_2_ value of the signal intensity in DNA microarray of CS (gray line, *y*-axis on the right side) was used as an estimate of the expression level for each gene. The percentage of upregulated (white bars) and downregulated (black bars) genes in ΔrpoZ in each expression category is shown. (**B**) Soluble proteins were isolated and fractionated to >100 kDa (contains RNAP core and holoenzyme) and 30–100 kDa fractions (free α and σ subunits). Equal amounts of fraction were loaded on SDS-PAGE gel, proteins were separated and the amounts of the α and SigA subunits were detected by western blotting. (**C**) A sequence logo of the promoter regions of genes downregulated in ΔrpoZ. (**D**) A sequence logo for genes upregulated in ΔrpoZ.

The findings that many downregulated genes encoded abundant proteins like photosynthetic proteins and that many upregulated genes belong to categories hypothetical or unknown (Figure 3) raised the question if deletion of the ω subunit affects differentially genes with high- and low-expression levels. Estimation of the gene expression level from the DNA microarray data showed that the most highly expressed genes of CS-encoded protein subunits of the light harvesting phycobilisome antenna, photosynthetic light reactions, ATP synthase, rubisco, carbon concentrating mechanisms, ribosomes, the nicotinamide adenine dinucleotide dehydrogenase (NDH) complex, the groES/EL chaperone and few others (see Supplementary Table S3 for a complete list of genes with log_2_ of the raw signal value >14, representing 3.8% of the genes). Interestingly, genes that were downregulated in ΔrpoZ mainly belonged to the highly expressed genes, whereas genes upregulated in ΔrpoZ showed only low or moderate expression in CS (Figure 4A).

Genes showing high expression in standard growth conditions can be assumed to be mainly transcribed by the primary σ factor SigA. To estimate the amount of SigA functioning in transcription, the distribution of SigA protein in the RNAP holoenzyme and in the free protein pool was measured. For this, soluble proteins were isolated and fractionated to >100 kDa fraction that contains RNAP complexes (>400 kDa) and to a 30–100 kDa fraction that contains free α subunits (41 kDa) and free sigA (60 kDa). The α subunit of RNAP was only found from the >100 kDa fraction, and similar quantities of α subunits in both strains confirmed that the same amount of RNAP complexes were present in ΔrpoZ and CS (Figure 4B). The SigA protein was found both in the >100 kDa (RNAP holoenzyme) and the 30–100 kDa (free SigA protein) fractions (Figure 4B). The SigA content of the RNAP holoenzyme in ΔrpoZ was only 79 ± 7% of that measured in CS, whereas free SigA was more abundant (171 ± 16%) in ΔrpoZ than in CS. The results suggest that the presence of the ω subunit favors the binding of the primary σ factor to the RNAP core, and downregulation of many highly expressed genes in ΔrpoZ might be due to the lowered SigA content of the RNAP holoenzyme. Further *in vivo* and *in vitro* experiments would be required to reveal why the absence of the ω subunit leads to a lower SigA content in RNAP holoenzyme.

To compare the promoter regions of upregulated and downregulated genes in ΔrpoZ, the −10 and −35 promoter regions were predicted for genes whose transcription initiation sites have been determined (15). The alignments and information contents of the promoter elements of upregulated and downregulated genes are shown in Supplementary Table S4. The sequence logos of promoter regions of genes that were downregulated in ΔrpoZ (Figure 4C) had a higher information content than those of upregulated genes (Figure 4D and Supplementary Table S4), although both had a highly conserved −10 region that resembles the −10 element of *E. coli* promoters (22). An extended −10 element, typical for highly expressed genes in *E. coli* (23), was found in the sequence logo of the downregulated genes (Figure 4C), suggesting that an extended −10 element is typical for highly expressed genes also in cyanobacteria. The −35 elements of upregulated and downregulated genes differed from each other (Figure 4C and D), and neither of them was similar to the *E. coli* −35 consensus sequence (22). The information content of the −35 region of downregulated genes was higher than that of the upregulated genes. An AT-rich sequence between the −10 and −35 elements was only detected in the sequence logo of genes that were downregulated in ΔrpoZ (Figure 4). The differences in the promoter elements of upregulated and downregulated genes further support the idea that transcriptional differences between CS and ΔrpoZ are caused by differences in σ factor recruitment.

Most genes showing upregulation in ΔrpoZ were weakly expressed in CS in standard conditions; expression of those genes may depend on the primary-like group 2 σ factors that closely resemble SigA but are non-essential in optimal conditions or on alternative group 3 σ factors that show considerable variation in amino acid sequence when compared with SigA. The *hspA* gene, recognized by SigB (24), and *pil* genes, recognized by SigF (25), provide examples of genes that are upregulated in ΔrpoZ and depend on a group 2 and a group 3 σ factor, respectively. Results from ΔrpoZ strains in different eubacteria support the role of the ω subunit as a factor influencing σ factor recruitment. In the *rpoZ* deletion mutant of *E. coli*, the primary σ factor σ^70^ is present in almost normal quantity but the RNAP largely recruits the group 2 σ factor σ^S^ instead of σ^70^, and DNA relaxation of ΔrpoZ strain can be suppressed by overexpressing σ^70^ in the ΔrpoZ strain (8). However, the interpretation of *E. coli* results is complicated because the inactivation of *rpoZ* decreases the expression of the downstream *spoT* gene affecting accumulation of (p)ppGpp (3). The (p)ppGpp, in turn, binds to RNAP core at the interface of β′ and ω (26) and facilitates recruitment of σ^S^ and enhances expression of stress responsive genes (27). Although ω-less *E. coli* strains are fully viable, some ω mutants are lethal, and *in vitro* studies showed that in one of the mutants transcription initiation was defective (28). The finding that cell surface properties change due to inactivation of the ω subunit in *M. smegmatis* and *S. coelicolor* (5,6) points to interplay between ω and σ subunits, as cell surface properties are known to be affected by alternative σ factors.

### Genes encoding proteins of ATP synthase, carbon concentrating mechanisms, CO_2_ fixation, NDH-1 and ATP synthase are downregulated in ΔrpoZ

The DNA microarray analysis revealed that the expression of several central photosynthetic protein complexes and biochemical pathways were downregulated in ΔrpoZ (Supplementary Table S2). All subunits of the ATP synthase were downregulated at the transcript level in ΔrpoZ, and downregulation of AtpE was verified at protein level as well (Figure 3B). The messenger RNA (mRNA) levels of *rbcS* and *rbcL* genes encoding the small and large subunit of rubisco, respectively, and the *rbcX* gene encoding rubisco activase were downregulated. Downregulation of rubisco large subunit was verified at protein level (Figure 3B). In addition to rubisco, many other genes encoding proteins of the carbon fixation pathway were downregulated. The central carboxysome operon *ccmK2K1LMN* (29) was downregulated but the carbonic anhydrase gene *ccaA* and the *ccmA*, *ccmK3*, *ccmK4* and *ccmO* genes were expressed similarly as in CS.

Different forms of the NDH-1 complex function either in CO_2_ uptake or in respiration and cyclic electron flow (30). All genes (*ndhA-C, E, G-K, M-N, S*), encoding NDH-1 core subunits (30,31), except *ndhO*, were downregulated in ΔrpoZ (Supplementary Table S2), and downregulation at protein level was verified for the NdhJ and NdhK proteins (Figure 3B). The *ndhF4* and *ndhD4* genes (but not *cupB*) for the CO_2_ uptake form of NDH-1 were downregulated, and also the other constitutively expressed low-affinity inorganic carbon transporter system, encoded by the *bicA* gene, was down. In addition, the *sbtAB* operon and *ndhF-ndhD3-cupA-cupS* operon, encoding low-CO_2_-inducible transporters of inorganic carbon, showed low expression in ΔrpoZ (Supplementary Table S2). Downregulation of *ndhD3* was verified at the protein level (Figure 3B). Furthermore, some subunits of the respiration and cyclic electron flow-specific form of NDH-1 and an operon including *ndhD6* and *ndhD5* genes were downregulated in ΔrpoZ (Supplementary Table S2).

Interestingly, downregulated genes included the genes *ccmR* (32) and *sll0822* (33), which encode repressor proteins for *sbt*, *ndhF3-ndhD3-cupA-cupS and mnh* (*slr2006-2010/ssr3409-3410/slr2011-2013*) operons, and *ccmR* and *ubiX* genes. Because both repressor protein genes and their target genes (except *ubiX*) were downregulated, absence of the ω subunit reveals a new common regulation system for a variety of genes involved in carbon concentrating and fixation.

### Genes upregulated in ΔrpoZ include many pili genes and the *hspA* heat shock gene

Genes upregulated in ΔrpoZ included mainly genes belonging to categories unknown or hypothetical in Cyanobase (Figure 3A). Many genes encoding pili proteins were upregulated in ΔrpoZ (Supplementary Table S1), and several genes encoding envelope components were downregulated (Supplementary Table S2). Despite the upregulation of many pili proteins, ΔrpoZ remained non-motile like our glucose tolerant host strain, CS. The visual appearance of ΔrpoZ colonies was similar to that of CS colonies but ΔrpoZ cells adhered more firmly to BG-11 plates and to walls of centrifuge tubes, suggesting that inactivation of the *rpoZ* gene causes changes in cell surface properties also in cyanobacteria.

In *E. coli,* inactivation of *rpoZ* increases the activity of the GroEL heat shock protein, and GroEL has been suggested to replace the chaperon activity of the ω subunit in the ΔrpoZ strain (34). In our ΔrpoZ strain, expression of *groEL* was not changed but instead the *hspA* heat shock gene was upregulated both at mRNA (Supplementary Table S1) and protein levels (Figure 3B).

### Physiological properties of ΔrpoZ

**Figure 5.**
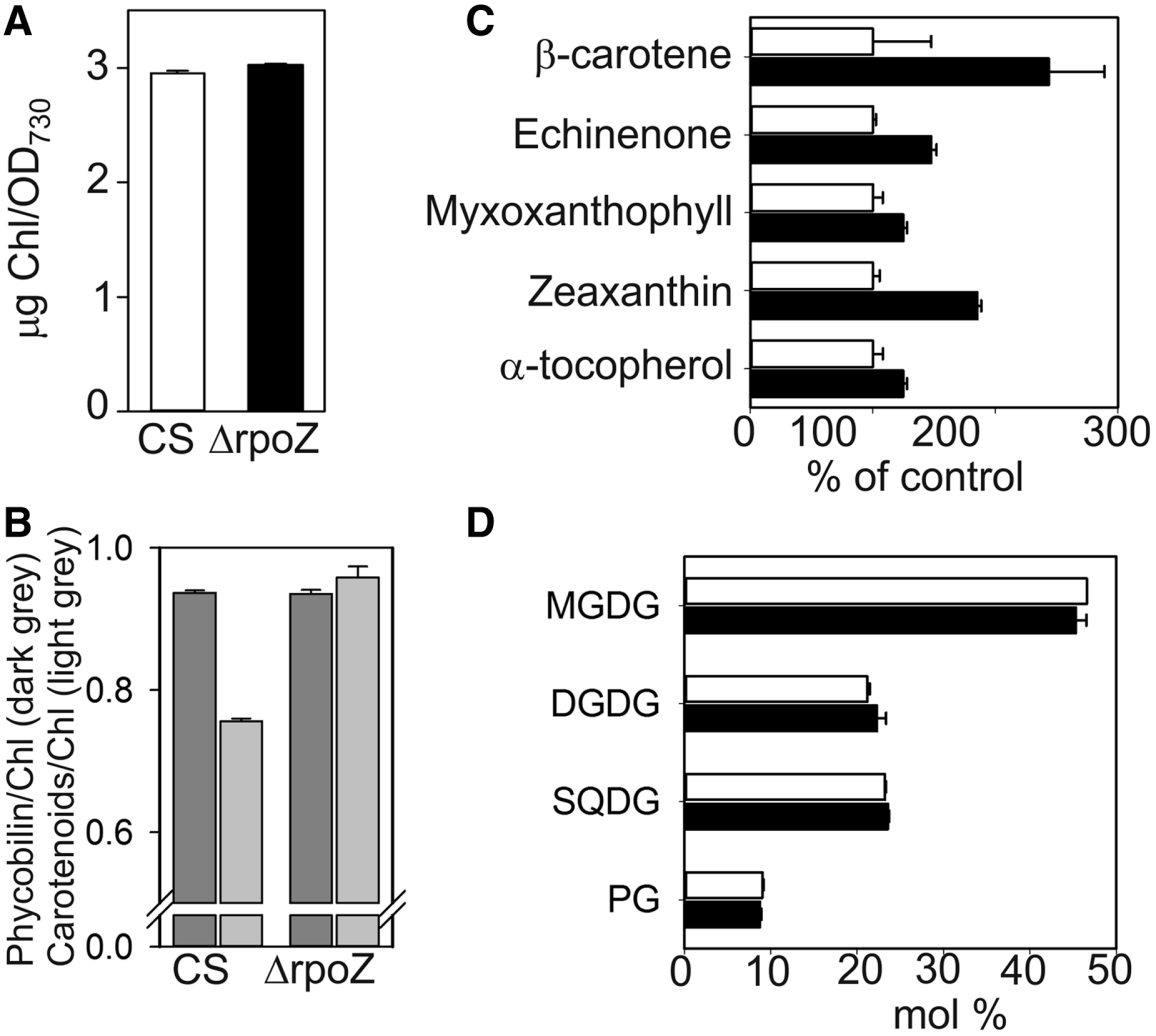
Pigment and lipid compositions of CS and ΔrpoZ in standard conditions. (**A**) Chl *a* content in 1 ml of cell culture (OD_730_ = 1). (**B**) The ratios of phycobilin (625 nm) to Chl *a* (678 nm) (dark gray bars) and carotenoids (485 nm) to Chl *a* (light gray bars) were calculated from *in vivo* absorption spectra. (**C**) Carotenoids were extracted with methanol and analyzed with HPLC, and the amount of each carotenoid in ΔrpoZ (black bars) was compared with that in CS (white bars). (**D**) Composition of membrane lipids in CS and ΔrpoZ. Each bar (A–D) represents an average of three independent experiments, and the error bars denote SE.

Cell cultures of the ΔrpoZ strain appeared yellowish, prompting us to analyze pigments. Chl *a* contents were similar in CS and ΔrpoZ (Figure 5A). *In vivo* absorption spectra revealed that the ratio of carotenoids to Chl *a* was high in the ΔrpoZ strain, whereas the ratio of phycobilisomes to Chl *a* was similar in ΔrpoZ and CS (Figure 5B), indicating that the high carotenoid content caused the yellowish appearance of ΔrpoZ cultures. HPLC analysis of carotenoids (Figure 5C) showed that the amounts of β-carotene and zeaxanthin were ∼2-fold higher in ΔrpoZ than in CS. The ΔrpoZ also contained high concentrations of the two other main carotenoids echinenone (1.5-fold) and myxoxanthopyll (1.25-fold). Membrane-bound carotenoids function as non-enzymatic antioxidants in cyanobacteria, mainly quenching and scavenging ^1^O_2_ (35). Another important ^1^O_2_ scavenger, α-tocopherol, was upregulated in ΔrpoZ as well (Figure 5C). The lipid composition was analyzed to find out whether the high carotenoid content of membranes is accompanied by changes in membrane lipids. Similar amounts of the main lipids, monogalactosyldiacylglycerol, digalactosyldiacylglycerol, sulfoquinovosyldiacylglycerol and phosphatidylglycerol were detected in ΔrpoZ and CS (Figure 5D). The fatty acid composition of lipids showed only some minor modifications in ΔrpoZ (Supplementary Figure S4).

**Figure 6.**
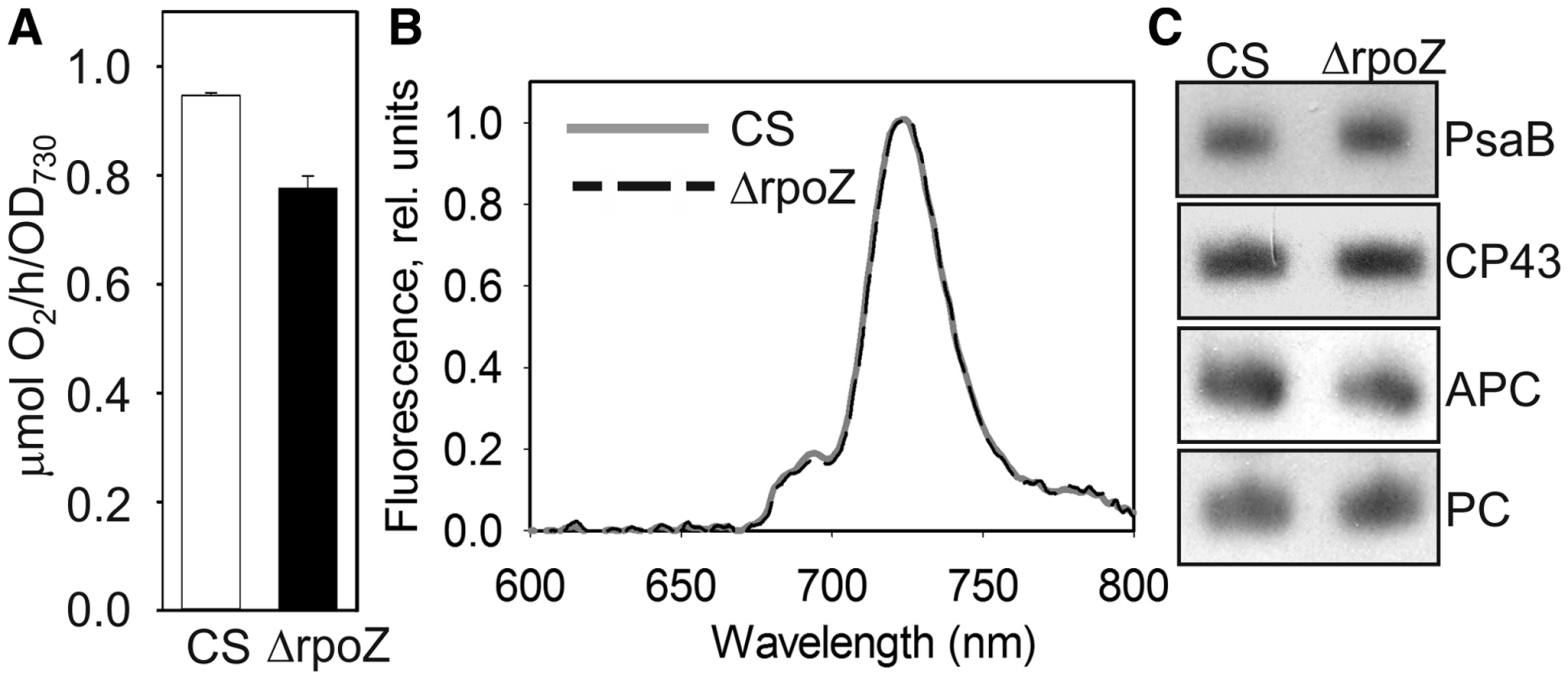
Photosynthetic properties of ΔrpoZ. (**A**) Light-saturated photosynthetic activity of a 1 ml of culture (OD_730_ = 1) of CS (white bar) and ΔrpoZ (black bar) in standard conditions. Each bar represents an average of three independent biological replicates, and the error bars denote SE. (**B**) Fluorescence at 77 K was measured using 440-nm light that excites Chl. The data were normalized by dividing with the height of the PSI emission peak at 723 nm. (**C**) Total proteins were isolated, separated with SDS-PAGE and the amounts of PSI reaction center protein PsaB, PSII core protein CP43 and the phycobilisome proteins allophycocyanin (APC) and phycocyanin (PC) were measured by western blotting. The protein contents of PsaB, CP43, ACP and PC were 99 ± 6%, 97 ± 7, 103 ± 7% and 99 ± 4, respectively, in ΔrpoZ of that measured in CS.

Although similar growth rates were measured for ΔrpoZ and CS, the light-saturated photosynthetic activity of ΔrpoZ was 20% lower than that of CS (Figure 6A), in accordance with a decreased rubisco content of ΔrpoZ (Figure 3B). Fluorescence emission spectra, measured at 77 K using Chl *a* excitation, were similar in ΔrpoZ and CS (Figure 6B), indicating a similar PSII:PSI ratio in both strains, and equal amounts of the PSI reaction center protein PsaB and the PSII core protein CP43 were detected in both strains (Figure 6C). The amounts of phycobilisome proteins, phycocyannin and allophycocyanin, were similar in both strains (Figure 6C). The mRNA levels encoding these light reaction proteins were also equal in ΔrpoZ and CS. Normal amounts of PSI and PSII and a reduced amount of rubisco might lead to imbalance of photosynthetic light reactions and carbon fixation, which in turn may increase the risk of production of reactive oxygen species (ROS). Lowered expression of the flavodiiron protein Flv3 [involved in a Mehler-like reaction that transfers electron from PSI to O_2_ without production of ROS (36)] in ΔrpoZ both at mRNA (Supplementary Table S3) and protein levels (Figure 3B) further increases the risk of ROS production. The *flv4-sll0218-flv2* operon, providing photoprotection for PSII (14,37), showed only low expression at the mRNA (Supplementary Table S2) and protein levels in ΔrpoZ (Figure 3B). Apparently, upregulation of carotenoids and α-tocopherol in ΔrpoZ provides protection against photoinhibition and adverse effects of ROS (14).

Our results show that the ω subunit is not an essential protein in cyanobacteria, in accordance with the results from other eubacteria. In cyanobacteria, the absence of the ω subunit has a negative effect on recruitment of the primary σ factor, which causes downregulation of highly expressed genes, and leads to a distinguished phenotype including low light-saturated photosynthetic activity and high carotenoid content of the mutant cells.

## SUPPLEMENTARY DATA

Supplementary Data are available at NAR Online.

## FUNDING

Academy of Finland. Funding for open access charge: Academy of Finland.

*Conflict of interest statement*. None declared.

## Supplementary Material

Supplementary Data
