# Supplementary material for: The omega subunit of the RNA polymerase core directs transcription efficiency in cyanobacteria
Source: Nucleic Acids Res. 2014 Jan 28;42(7):4606–14. doi: 10.1093/nar/gku084 (PMC3985657; doi:10.1093/nar/gku084)
Supplement: Supplementary Data [file supp_42_7_4606__index.html]

The omega subunit of the RNA polymerase core directs transcription efficiency in cyanobacteria — The omega subunit of the RNA polymerase core directs transcription efficiency in cyanobacteria — Supplementary Data 

# The omega subunit of the RNA polymerase core directs transcription efficiency in cyanobacteria

## Supplementary Data

files

**Files in this Data Supplement:**

- Supplementary Data - pdf file
