# Supplementary material for: The omega subunit of the RNA polymerase core directs transcription efficiency in cyanobacteria
Source: Nucleic Acids Res. 2014 Jan 28;42(7):4606–14. doi: 10.1093/nar/gku084 (PMC3985657; doi:10.1093/nar/gku084)
Supplement: Supplementary Data [file supp_gku084_nar-03376-r-2013-File008.pdf]

Supplemental Information

The omega subunit of the RNA polymerase core directs transcription efficiency in cyanobacteria

Liisa Gunnelius, Kaisa Hakkila, Juha Kurkela, Hajime Wada, Esa Tyystjärvi, and Taina Tyystjärvi

Figure S1

Figure S2

Figure S3

Figure S4

Table S1

Table S2

Table S3

Table S4

Supplemental References

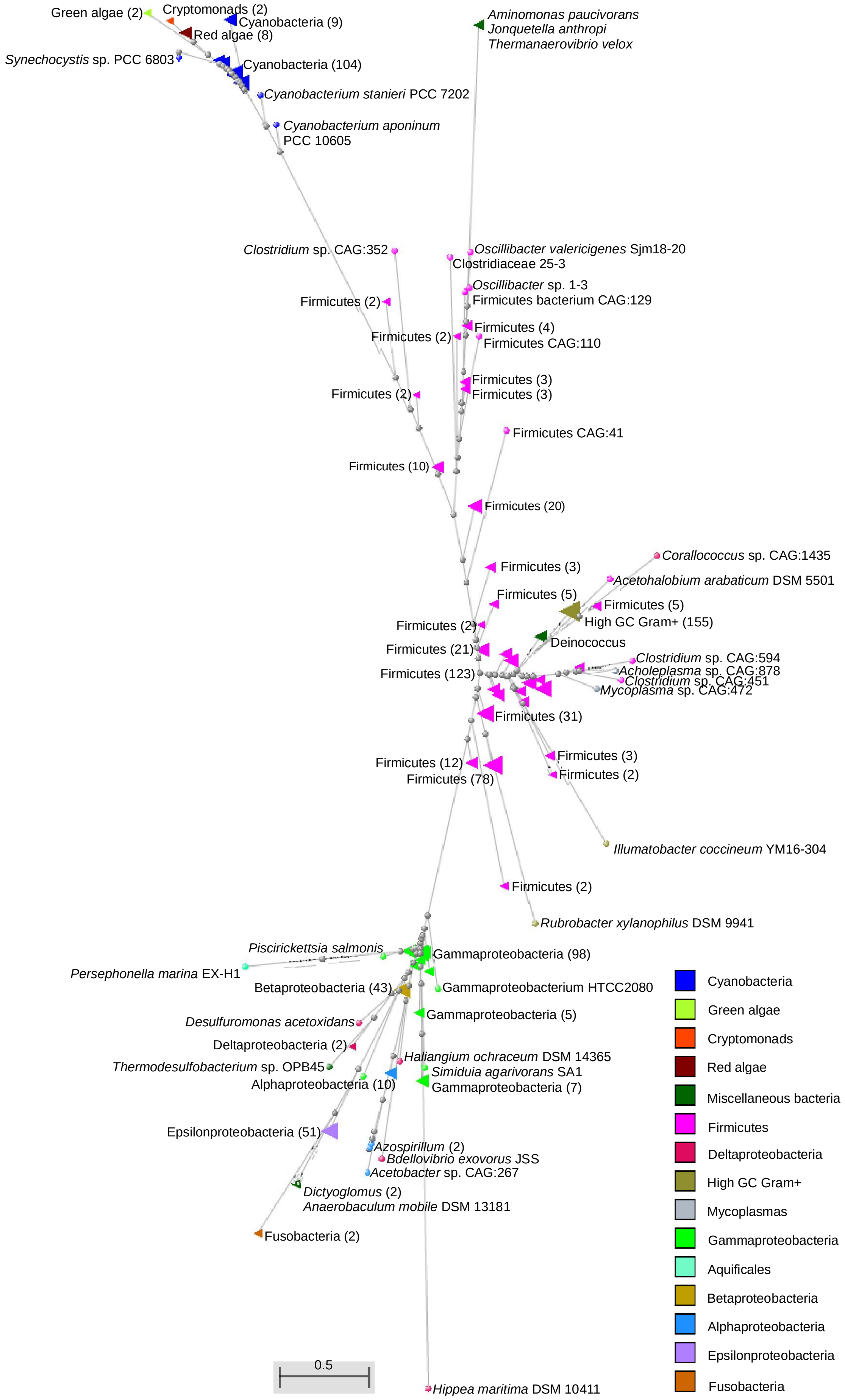

Figure S1. A phylogenetic tree of ω subunits. The NCBI database of non-redundant protein sequences was queried with the sequence of the ω subunit of *Synechocystis* sp. PCC 6803 (RefSeq ID: NP_441766.1) with DELTA-BLAST (1) using BLOSUM80 matrix. From the result list, only bacterial and plastid sequences annotated as the ω subunit with E-values better than the threshold 0.005 were selected and a distance tree was generated with default settings. Colored triangles depict closely clustered sequences from different strains, the number of strains is indicated in brackets, and colored spheres denote single strains.

```
                2048110       120       130       140       150       160       170       180       190       200
        ....|....|....|....|....|....|....|....|....|....|....|....|....|....|....|....|....|....|....|....|
Kazusa  TAACGTCAGGGGAAAAAGGGGCTTAAACATGGAACTGGGGGAATTAATTATCGCTAATAATTTCTGGGCGGGTGAGCTCGTCGGACATTTCAATGATGGC
GT-S    TAACGTCAGGGGAAAAAGGGGCTTAAACATGGAACTGGGGGAATTAATTATCGCTAATAATTTCTGGGCGGGTGAGCTCGTCGGACATTTCAATGATGGC
GT-I    TAACGTCAGGGGAAAAAGGGGCTTAAACATGGAACTGGGGGAATTAATTATCGCTAATAATTTCTGGGCGGGTGAGCTCGTCGGACATTTCAATGATGGC
GT-M    TAACGTCAGGGGAAAAAGGGGCTTAAACATGGAACTGGGGGAATTAATTATCGCTAATAATTTCTGGGCGGGTGAGCTCGTCGGACATTTCAATGATGGC
CS      TAACGTCAGGGGAAAAAGGGGCTTAAACATGGAACTGGGGGAATTAATTATCGCTAATAATTTCTGGGCGGGTGAGCTCGTCGGACATTTCAATGATGGC

                2048210       220       230       240       250       260       270       280       290       300
        ....|....|....|....|....|....|....|....|....|....|....|....|....|....|....|....|....|....|....|....|
Kazusa  CCGGATAGCCGGCTTCATGTTGGGATCGTCAATGCTGTCAAAATCTTCGGAGCGATTTTCCTTGGCTCGCTTGGCTACCCTGACAGTAATGTTGTAGCGG
GT-S    CCGGATAGCCGGCTTCATGTTGGGATCGTCAATGCTGTCAAAATCTTCGGAGCGATTTTCCTTGGCTCGCTTGGCTACCCTGACAGTAATGTTGTAGCGG
GT-I    CCGGATAGCCGGCTTCATGTTGGGATCGTCAATGCTGTCAAAATCTTCGGAGCGATTTTCCTTGGCTCGCTTGGCTACCCTGACAGTAATGTTGTAGCGG
GT-M    CCGGATAGCCGGCTTCATGTTGGGATCGTCAATGCTGTCAAAATCTTCGGAGCGATTTTCCTTGGCTCGCTTGGCTACCCTGACAGTAATGTTGTAGCGG
CS      CCGGATAGCCGGCTTCATGTTGGGATCGTCAATGCTGTCAAAATCTTCGGAGCGATTTTCCTTGGCTCGCTTGGCTACCCTGACAGTAATGTTGTAGCGG

                2048310       320       330       340       350       360       370       380       390       400
        ....|....|....|....|....|....|....|....|....|....|....|....|....|....|....|....|....|....|....|....|
Kazusa  TTTGATGCGGCTCCTAGGAGCTCTTCGGAACGATAAATAATGTGGTTAGAATCCAAATTACTACGCTTGGTCATGGTAAACAGGGTAAGGGGGACAATAT
GT-S    TTTGATGCGGCTCCTAGGAGCTCTTCGGAACGATAAATAATGTGGTTAGAATCCAAATTACTACGCTTGGTCATGGTAAACAGGGTAAGGGGGACAATAT
GT-I    TTTGATGCGGCTCCTAGGAGCTCTTCGGAACGATAAATAATGTGGTTAGAATCCAAATTACTACGCTTGGTCATGGTAAACA------------------
GT-M    TTTGATGCGGCTCCTAGGAGCTCTTCGGAACGATAAATAATGTGGTTAGAATCCAAATTACTACGCTTGGTCATGGTAAACA------------------
CS      TTTGATGCGGCTCCTAGGAGCTCTTCGGAACGATAAATAATGTGGTTAGAATCCAAATTACTACGCTTGGTCATGGTAAACAGGGTAAGGGGGACAATAT

                2048410   2049580       590       600       610       620       630       640       650       660
        ....|....|....|....|....|....|....|....|....|....|....|....|....|....|....|....|....|....|....|....|
Kazusa  GGCGATCGCC slr1635 TCTGGGCGATCGCCATTCGGAAAAGCAAAAAAGCAGAAACCGGGTGGAAGAAAAATCCGCAGAATACGCTTCCCAGTCTAG
GT-S    GGCGATCGCC slr1635 TCTGGGCGATCGCCATTCGGAAAAGCAAAAAAGCAGAAACCGGGTGGAAGAAAAATCCGCAGAATACGCTTCCCAGTCTAG
GT-I    -----------------------GGCGATCGCCATTCGGAAAAGCAAAAAAGCAGAAACCGGGTGGAAGAAAAATCCGCAGAATACGCTTCCCAGTCTAG
GT-M    -----------------------GGCGATCGCCATTCGGAAAAGCAAAAAAGCAGAAACCGGGTGGAAGAAAAATCCGCAGAATACGCTTCCCAGTCTAG
CS      -----------------------GGCGATCGCCATTCGGAAAAGCAAAAAAGCAGAAACCGGGTGGAAGAAAAATCCGCAGAATACGCTTCCCAGTCTAG

                2049670       680       690       700       710       720       730       740       750       760
        ....|....|....|....|....|....|....|....|....|....|....|....|....|....|....|....|....|....|....|....|
Kazusa  CGGAAAATTACCTCGGCTCCATCCCAACTGACCTAATATTGACCCTCCCCTCTATCGACATATCGACAGTGCATCCATTAACCATGGACGAAAATATCGG
GT-S    CGGAAAATTACCTCGGCTCCATCCCAACTGACCTAATATTGACCCTCCCCTCTATCGACATATCGACAGTGCATCCATTAACCATGGACGAAAATATCGG
GT-I    CGGAAAATTACCTCGGCTCCATCCCAACTGACCTAATATTGACCCTCCCCTCTATCGACATATCGACAGTGCATCCATTAACCATGGACGAAAATATCGG
GT-M    CGGAAAATTACCTCGGCTCCATCCCAACTGACCTAATATTGACCCTCCCCTCTATCGACATATCGACAGTGCATCCATTAACCATGGACGAAAATATCGG
CS      CGGAAAATTACCTCGGCTCCATCCCAACTGACCTAATATTGACCCTCCCCTCTATCGACATATCGACAGTGCATCCATTAACCATGGACGAAAATATCGG
```

Figure S2. Sequence comparison of the *rpoZ* gene and surrounding areas in different laboratory strains of *Synechocystis* sp. PCC 6803. The sequences include the originally sequenced Kazusa strain (2), the re-sequenced Kazusa strain (GT-S), a glucose tolerant strain from Ikeuchi laboratory (GT-I) (3), a motile, glucose tolerant strain from Shestakov laboratory (GT-M) (4) and our glucose tolerant non-motile strain (CS). The *rpoZ* gene (*ssl2892*) is orange, the end parts of *sll1532* and *slr1636*, encoding hypothetical proteins, are blue and green, respectively. The location of the transposase *slr1635* is indicated with grey and the transposase related palindromic repeats are pink.

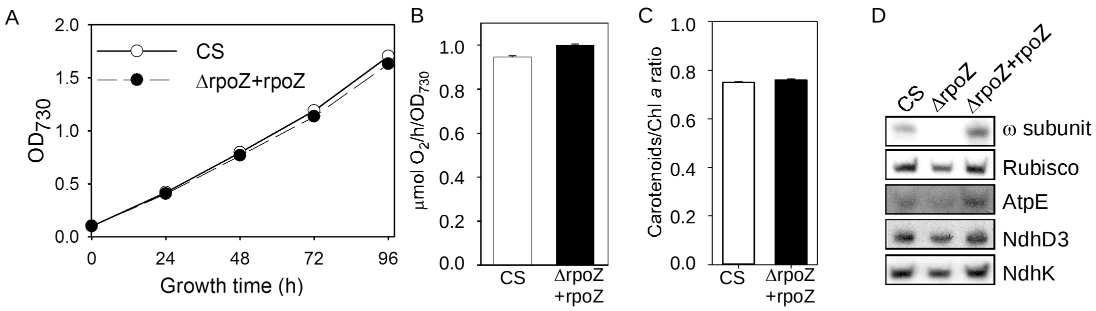

Figure S3. Properties of the complementation strain. **(A)** Growth of the control (CS) and complementation (ΔrpoZ+rpoZ) strains in standard conditions. **(B)** Light-saturated photosynthetic activity. **(C)** Ratio of carotenoids to Chl *a* measured from *in vivo* absorption spectra. **(D)** Western blots showing ω, Rubisco large subunit, AtpE, NdhD3 and NdhK contents in CS, ΔrpoZ and ΔrpoZ+rpoZ. The error bars denote SE and are shown if bigger than the symbol.

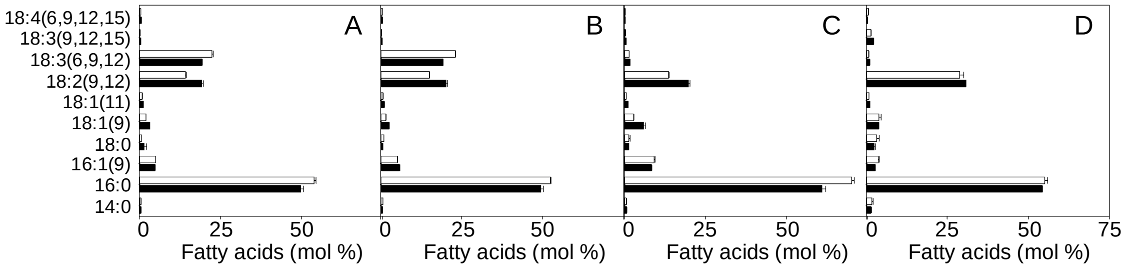

Figure S4. Fatty acid composition of MGDG **(A)**, DGDG **(B)**, SQDG **(C)** and PG **(D)** in the control (CS) and ΔrpoZ strains.

Table S1. Genes that are at least two-fold up-regulated in ΔrpoZ compared to the control strain (log_2 of fold change ≥1.00 and P-value <0.05) except that all genes encoding pilin proteins are shown.

Category and function	Gene	ORF	FC (log_2)	P-value
Amino acid biosynthesis				
Nitrilase	*merR*	*sll0784*	1.30	0.003575
cysteine desulfurase NifS	*nifS1*	*slr0387*	1.58	0.001962
glutamate--ammonia ligase	*glnN*	*slr0288*	1.42	0.035451
NADH-dependent glutamate synthase large subunit	*gltB*	*sll1502*	1.54	0.000048
Biosynthesis of cofactors, prosthetic groups, and carriers				
light-independent protochlorophyllide reductase subunit ChlB	*chlB*	*slr0772*	2.29	0.001552
light-independent protochlorophyllide reductase iron protein subunit ChlL	*chlL*	*slr0749*	1.33	0.001692
thioredoxin	*trxA*	*slr0623*	2.07	0.001410
Cellular processes				
16.6 kDa small heat shock protein, molecular chaperone	*hspA*	*sll1514*	1.92	0.000001
thiophen and furan oxidation protein	*trmE*	*sll1615*	1.09	0.018389
Pilin proteins				
pilin polypeptide PilA1	*pilA1*	*sll1694*	0.66	0.024896
pilin polypeptide PilA2	*pilA2*	*sll1695*	1.00	0.005302
putative TatA protein	*pilA3*	*slr1046*	-0.45	0.005783
type 4 pilin-like protein, or general secretion pathway protein G	*pilA4*	*slr1456*	0.22	0.254020
type 4 pilin-like protein	*pilA5*	*slr1928*	1.69	0.015133
type 4 pilin-like protein	*pilA6*	*slr1929*	1.56	0.002666
type 4 pilin-like protein	*pilA7*	*slr1930*	0.26	0.251914
type 4 pilin-like protein	*pilA8*	*slr1931*	0.04	0.780298
type 4 pilin-like protein, essential for motility	*pilA9*	*slr2015*	0.64	0.180344
type 4 pilin-like protein, essential for motility	*pilA10*	*slr2016*	-0.07	0.722345
type 4 pilin-like protein, essential for motility	*pilA11*	*slr2017*	-0.13	0.604387
pilus biogenesis protein homologous to general secretion pathway protein E	*pilB1*	*slr0063*	0.32	0.106016
probable general secretion pathway protein E	*pilB2*	*slr0079*	0.07	0.658998
a part of pilC, pilin biogenesis protein, required for twitching motility	*pilC*	*slr0162*	1.46	0.000410
a part of pilC, pilin biogenesis protein, required for twitching motility	*pilC'*	*slr0163*	1.06	0.000076
type 4 prepilin-like proteins leader peptide processing enzyme	*pilD*	*slr1120*	-0.04	0.830855
similar to chemotaxis protein CheW	*pilI*	*slr1043*	-0.15	0.325201
methyl-accepting chemotaxis protein, biogenesis of thick pilli	*pilJ, ctr1*	*slr1044*	-0.38	0.111543
two-component response regulator PatA subfamily	*pilG, rre6*	*slr1041*	-0.01	0.922585
two-component response regulator CheY subfamily	*pilH, rre7*	*slr1042*	0.61	0.001545
probable fimbrial assembly protein PilM, required for motility	*pilM*	*slr1274*	-0.31	0.093117
hypothetical protein	*pilN*	*slr1275*	-0.95	0.013005
hypothetical protein	*pilO*	*slr1276*	-1.29	0.001112
pilus assembly protein homologous to general secretion pathway protein D	*pilQ*	*slr1277*	-1.36	0.001350
twitching motility protein PilT	*pilT1*	*slr0161*	1.58	0.000084
twitching mobility protein	*pilT2*	*sll1533*	1.96	0.000008
type IV pilus biogenesis protein PilI homolog		*sll1107*	0.24	0.188156

Table S1. *continued*

Category and function	Gene	ORF	FC (log_2)	P-value
Central intermediary metabolism				
neopullulanase	*nplT*	*sll0842*	1.23	0.000109
glycogen phosphorylase	*glgP*	*sll1356*	1.06	0.001545
Energy metabolism				
carbamate kinase	*arcC*	*sll0573*	2.27	0.002451
phosphofructokinase	*pfkB1*	*sll1196*	1.52	0.000346
phosphotransacetylase	*pta*	*slr2132*	1.20	0.003062
phosphoenolpyruvate synthase	*ppsA*	*slr0301*	3.94	0.000252
acetyl-coenzyme A synthetase	*acs*	*sll0542*	1.48	0.000004
Fatty acid, phospholipid and sterol metabolism				
similar to biotin [acetyl-CoA-carboxylase] ligase	*birA*	*sll1655*	2.17	0.000057
Photosynthesis and respiration				
photosystem I subunit IV	*psaE*	*ssr2831*	1.72	0.003538
photosystem II 13 kDa protein homolog	*psb28-2*	*slr1739*	1.12	0.001784
pyruvate flavodoxin oxidoreductase	*nifJ*	*sll0741*	2.48	0.000113
Regulatory functions				
cyanobacterial phytochrome 1, two-component sensor histidine kinase	*hik35*	*slr0473*	1.78	0.000579
positive phototaxis protein, two-component response regulator PatA subfamily	*pixG*	*sll0038*	1.33	0.000126
probable transcriptional regulator	*dnr*	*slr0449*	1.38	0.001241
transcriptional regulator	*pfsR*	*sll1392*	1.09	0.000885
transcriptional regulator		*sll1286*	1.01	0.010826
two-component hybrid sensor and regulator	*hik21*	*slr2098*	1.01	0.003093
two-component response regulator CheY subfamily	*rre27*	*slr0474*	2.08	0.000146
two-component response regulator NarL subfamily	*rre30*	*sll0485*	1.05	0.007034
two-component response regulator OmpR subfamily	*rre34*	*sll0789*	1.00	0.000565
two-component sensor histidine kinase	*hik13*	*sll1003*	1.37	0.001392
two-component transcription regulator OmpR subfamily	*rre38*	*slr1584*	1.34	0.006272
DNA replication, restriction, modification, recombination, and repair				
formamidopyrimidine-DNA glycosylase	*mutM*	*slr1689*	1.50	0.000471
Transcription				
RNA polymerase ECF-type (group 3) sigma-E factor	*sigH*	*sll0856*	1.31	0.008649
Translation				
tRNA (guanine-N1)-methyltransferase	*trmD*	*sll1198*	3.61	0.000041
peptidyl-tRNA hydrolase	*PTH*	*slr0922*	1.26	0.000913
elongation factor EF-G	*fus*	*sll0830*	2.34	0.000043
Transport and binding proteins				
ABC transport system permease protein		*sll1081*	1.45	0.001890
low affinity sulfate transporter		*slr0096*	1.25	0.004389
Mn transporter MntC	*mntC*	*sll1598*	1.21	0.000838
probable sodium/calcium exchanger protein		*slr0681*	2.03	0.000014
ferric aerobactin receptor, FhuA homolog	*iutA*	*sll1206*	1.48	0.001097

Table S1. *continued*

Category and function	Gene	ORF	FC (log_2)	P-value
probable polysaccharide ABC transporter permease protein	*rfbA*	*slr2107*	1.05	0.001202
glucose transport protein	*glcP*	*sll0771*	1.34	0.000976
iron transport system substrate-binding protein	*futA1*	*slr1295*	1.21	0.001934
probable polysaccharide ABC transporter ATP binding subunit	*rfbB*	*slr2108*	1.12	0.019317
Other categories				
light repressed protein A homolog	*lrtA*	*sll0947*	2.18	0.000039
stress induced hydrophobic peptide homolog	*ESI3*	*ssr1169*	1.18	0.003802
bacterioferritin-associated ferredoxin		*ssl2250*	1.15	0.032240
4-hydroxybutyrate coenzyme A transferase.		*slr1888*	1.24	0.000003
probable glycosyltransferase	*rfbZ*	*sll1534*	1.82	0.000934
putative diaphorase subunit of the bidirectional hydrogenase	*hoxE*	*sll1220*	1.37	0.000407
diaphorase subunit of the bidirectional hydrogenase	*hoxF*	*sll1221*	1.22	0.000459
putative hydrogenase expression/formation protein HypA1	*hypA1*	*slr1675*	1.16	0.016503
putative transposase [ISY352e: 2921301 - 2921595, join 3108631 - 3109754]	*ISY352e2*	*sll0668*	1.37	0.002513
putative transposase [ISY802a: 852462 - 853369]	*ISY802a*	*sll1791*	1.42	0.001033
putative transposase [ISY802a: 852462 - 853369]	*ISY802a*	*sll1792*	1.23	0.000305
putative transposase [ISY523e: 2441031 - 2441901]	*ISY523e*	*slr0350*	1.12	0.003375
putative transposase [ISY802c: 3066278 - 3067184]	*ISY802c*	*slr0799*	1.33	0.001764
putative transposase [ISY802c: 3066278 - 3067184]	*ISY802c*	*slr0800*	1.63	0.000129
putative transposase		*slr1523*	1.50	0.002165
putative transposase		*sml0010*	1.14	0.015913
putative transposase [ISY802b(partial copy): 1384736 - 1385513]	*ISY802b*	*ssr2078*	1.84	0.000118
putative transposase		*ssr2227*	1.93	0.001383
Hypothetical				
hypothetical protein		*sll0023*	1.14	0.008932
hypothetical protein		*sll0198*	1.81	0.001560
hypothetical protein		*sll0253*	2.45	0.000524
hypothetical protein		*sll0283*	2.31	0.000049
periplasmic protein, function unknown		*sll0314*	1.11	0.000012
hypothetical protein		*sll0335*	1.11	0.011390
hypothetical protein		*sll0381*	1.20	0.027722
hypothetical protein		*sll0382*	1.04	0.007387
hypothetical protein		*sll0412*	1.63	0.000223
hypothetical protein		*sll0532*	1.31	0.000564
hypothetical protein		*sll0543*	1.06	0.001687
hypothetical protein		*sll0572*	1.26	0.033000
hypothetical protein YCF35	*ycf35*	*sll0661*	1.26	0.000029
hypothetical protein		*sll0742*	1.01	0.008513
hypothetical protein		*sll0788*	1.20	0.000933
hypothetical protein		*sll0997*	1.25	0.001430
hypothetical protein		*sll1191*	1.05	0.003134

Table S1. *continued*

Category and function	Gene	ORF	FC (log_2)	P-value
hypothetical protein		*sll1222*	1.10	0.000281
hypothetical protein		*sll1247*	1.50	0.000629
hypothetical protein		*sll1500*	1.33	0.008080
hypothetical protein		*sll1651*	1.01	0.016290
hypothetical protein	*usp*	*sll1654*	2.15	0.000029
hypothetical protein		*sll1658*	1.29	0.015988
hypothetical protein		*slr0144*	3.83	0.000011
hypothetical protein		*slr0146*	2.65	0.000092
hypothetical protein		*slr0147*	2.45	0.000183
hypothetical protein	*fdx*	*slr0148*	1.72	0.001352
hypothetical protein		*slr0217*	1.34	0.000538
hypothetical protein	*usp1*	*slr0244*	1.12	0.000074
hypothetical protein		*slr0249*	1.46	0.000051
hypothetical protein	*syn-lov*	*slr0359*	1.01	0.000737
hypothetical protein		*slr0459*	1.55	0.000497
hypothetical protein		*slr0680*	2.16	0.000161
hypothetical protein		*slr0885*	1.11	0.007696
hypothetical protein		*slr0888*	3.11	0.000268
hypothetical protein		*slr0889*	1.33	0.000100
hypothetical protein		*slr0921*	1.36	0.000445
hypothetical protein	*rfrP*	*slr0967*	1.80	0.010371
hypothetical protein	*rfrK*	*slr1152*	2.33	0.001134
hypothetical protein		*slr1259*	1.26	0.000196
hypothetical protein		*slr1327*	1.07	0.000854
hypothetical protein		*slr1438*	2.84	0.000017
hypothetical protein		*slr1535*	1.36	0.000719
hypothetical protein		*slr1704*	3.56	0.000422
hypothetical protein		*slr1811*	1.41	0.002237
hypothetical protein		*slr1812*	1.31	0.005096
hypothetical protein		*slr1813*	1.32	0.001965
hypothetical protein		*slr1814*	1.44	0.001489
hypothetical protein		*slr1815*	1.43	0.001733
hypothetical protein	*rfrH*	*slr1851*	3.74	0.000065
hypothetical protein	*hoxW*	*slr1876*	1.38	0.000517
hypothetical protein		*slr1957*	2.20	0.000888
hypothetical protein		*slr2000*	1.19	0.000043
putative PP2C-type protein phosphatase	*rsbU*	*slr2031*	1.47	0.000521
hypothetical protein		*slr2052*	1.40	0.001860
hypothetical protein		*ssl0832*	1.16	0.012850
hypothetical protein		*ssl1004*	1.07	0.001148
hypothetical protein		*ssl1046*	1.06	0.000961

Table S1. *continued*

Category and function	Gene	ORF	FC (log_2)	P-value
hypothetical protein		*ssl1263*	1.27	0.000190
hypothetical protein		*ssl3573*	2.01	0.000590
hypothetical protein		*ssr0755*	1.32	0.000728
hypothetical protein		*ssr1251*	1.74	0.001915
hypothetical protein		*ssr1375*	1.71	0.000244
hypothetical protein		*ssr1499*	1.39	0.002704
hypothetical protein	*pgr5*	*ssr2016*	1.68	0.000496
hypothetical protein		*ssr2708*	1.36	0.006220
hypothetical protein		*ssr2723*	1.26	0.001157
hypothetical protein		*ssr2802*	1.31	0.015511
hypothetical protein		*ssr3154*	1.19	0.000424
Unknown				
unknown protein		*sll0022*	1.14	0.002978
unknown protein		*sll0024*	1.53	0.006092
unknown protein		*sll0243*	1.16	0.003506
unknown protein		*sll0376*	2.81	0.000097
unknown protein		*sll0441*	1.70	0.001791
unknown protein		*sll0539*	1.17	0.008011
unknown protein		*sll0783*	1.46	0.007104
unknown protein		*sll0857*	1.24	0.014025
unknown protein		*sll1236*	2.07	0.000191
unknown protein		*sll1241*	1.01	0.034357
unknown protein		*sll1396*	1.07	0.001119
unknown protein		*sll1426*	1.22	0.000036
unknown protein		*sll1476*	1.02	0.035798
unknown protein	*ligA*	*sll1583*	1.67	0.000030
unknown protein		*sll1764*	1.01	0.000027
unknown protein		*sll1862*	1.02	0.004612
unknown protein		*sll1863*	1.01	0.010581
unknown protein		*sll1882*	1.31	0.001492
unknown protein		*slr0112*	1.10	0.000236
unknown protein		*slr0145*	3.36	0.000004
unknown protein		*slr0226*	2.79	0.000215
unknown protein		*slr0271*	2.34	0.000013
unknown protein		*slr0439*	1.25	0.015691
unknown protein		*slr0579*	2.13	0.000510
unknown protein		*slr0581*	1.21	0.001887
unknown protein		*slr0582*	1.28	0.000272
unknown protein		*slr0587*	2.27	0.000105
periplasmic protein, function unknown		*slr0841*	1.01	0.000586
unknown protein		*slr0890*	1.20	0.002902

Table S1. *continued*

Category and function	Gene	ORF	FC (log_2)	P-value
unknown protein		*slr1232*	1.47	0.002652
unknown protein		*slr1437*	3.35	0.000006
unknown protein		*slr1475*	1.14	0.000791
hypothetical protein (target gene of sycrp1)	*cccS*	*slr1667*	2.82	0.002603
unknown protein		*slr1670*	1.44	0.000058
unknown protein		*slr1681*	1.09	0.017573
unknown protein		*slr1726*	1.03	0.001072
unknown protein		*slr1768*	1.14	0.002388
unknown protein		*slr1862*	3.62	0.000386
unknown protein		*slr1958*	2.15	0.000383
unknown protein		*ssl0318*	1.74	0.000792
unknown protein		*ssl2384*	4.32	0.000004
unknown protein		*ssl2891*	1.95	0.001659
unknown protein		*ssr0511*	1.04	0.038808
unknown protein		*ssr0693*	1.86	0.000517
unknown protein		*ssr2153*	1.28	0.004508
unknown protein		*ssr2194*	3.61	0.000112
unknown protein		*ssr2201*	1.20	0.006471
unknown protein		*ssr2333*	1.08	0.005828
unknown protein		*ssr3129*	3.76	0.000083

Table S2. Gene at least two-fold down-regulated in ΔrpoZ compared to the control strain (log_2 of fold change ≤-1.00 and a P-value <0.05) except that all genes for ATP synthase, CO_2 concentrating mechanism, Rubisco, NDH-1 complex, *sbt* and *mnh* operons and regulators controlling expression of low carbon inducible genes are all shown.

Category and function	Gene	ORF	FC (log_2)	P-value
Amino acid biosynthesis				
threonine synthase	*thrC*	*sll1688*	-1.99	0.000030
ferredoxin-nitrate reductase	*narB*	*sll1454*	-2.02	0.001115
ferredoxin-dependent glutamate synthase	*glsF*	*sll1499*	-1.33	0.001938
cysteine desulfurase	*nifS*	*slr0077*	-1.62	0.008349
Biosynthesis of cofactors, prosthetic groups, and carriers				
riboflavin biosynthesis protein RibA	*ribA*	*sll1894*	-1.15	0.005313
glutamate-1-semialdehyde aminomutase	*hemL*	*sll0017*	-1.09	0.001990
ferrochelatase	*hemH*	*slr0839*	-1.19	0.000009
pyridine nucleotide transhydrogenase beta subunit	*pntB*	*slr1434*	-1.11	0.021472
probable glucosyl transferase		*slr1125*	-1.05	0.004190
phytoene dehydrogenase (phytoene desaturase)	*crtP*	*slr1254*	-1.80	0.004324
glutathione synthetase	*gshB*	*slr1238*	-1.06	0.010862
Cell envelope				
putative peptidase	*nlpD*	*slr0993*	-1.02	0.009465
phospho-N-acetylmuramoyl-pentapeptide-transferase	*mraY*	*sll0657*	-1.01	0.005327
N-acetylmuramoyl-L-alanine amidase, periplasmic protein	*amiA, amiC*	*slr1744*	-1.01	0.000509
UDP-N-acetylglucosamine 1-carboxyvinyltransferase	*murA*	*slr0017*	-1.42	0.000047
glutamate racemase	*mull, murl*	*slr1746*	-1.53	0.000023
UDP-3-0-acyl N-acetylglcosamine deacetylase	*envA, lpxC*	*sll1508*	-1.12	0.000791
GDP-fucose synthetase	*wcaG*	*sll1213*	-1.51	0.001521
Cellular processes				
cell division protein FtsH	*ftsH2*	*slr0228*	-1.00	0.000105
HtpG, heat shock protein 90, molecular chaperone	*htpG*	*sll0430*	-1.22	0.022462
superoxide dismutase	*sodB*	*slr1516*	-1.01	0.000645
pilus assembly protein homologous to general secretion pathway protein D	*pilQ*	*slr1277*	-1.36	0.001350
methyl-accepting chemotaxis protein	*taxD2*	*sll1294*	-1.73	0.000170
Central intermediary metabolism				
glycogen operon protein GlgX homolog	*glgX*	*slr0237*	-1.05	0.010640
isoamylase	*glgX*	*slr1857*	-1.49	0.001136
1,4-alpha-glucan branching enzyme	*glgB*	*sll0158*	-1.04	0.000693
phosphoglucomutase	*pgm*	*sll0726*	-1.31	0.008735
soluble inorganic pyrophosphatase	*ppa*	*slr1622*	-1.07	0.000275
Energy metabolism				
asparaginase		*sll0422*	-1.26	0.012872
glycine dehydrogenase	*gcvP*	*slr0293*	-2.68	0.000020
carbamoyl-phosphate synthase, pyrimidine-specific, large chain	*pyrA*	*sll0370*	-1.54	0.000598

Table S2. *continued*

Category and function	Gene	ORF	FC (log_2)	P-value
adenosylhomocysteinase	*ahcY*	*sll1234*	-1.35	0.001297
phosphoglycerate mutase	*gpmB*	*slr1124*	-1.34	0.001804
enolase	*eno*	*slr0752*	-1.07	0.001554
phosphoglycerate kinase	*pgk*	*slr0394*	-2.16	0.000001
fructose-bisphosphate aldolase, class II	*fbaA*	*sll0018*	-1.43	0.000140
glucose 6-phosphate dehydrogenase	*zwf*	*slr1843*	-1.18	0.008704
pentose-5-phosphate-3-epimerase	*rpe*	*sll0807*	-1.05	0.001518
GDP-mannose 4,6-dehydratase	*gmd*	*sll1212*	-1.59	0.000110
Fatty acid, phospholipid and sterol metabolism				
acyl-lipid desaturase (delta 12)	*desA*	*slr1350*	-1.65	0.001543
acyl-lipid desaturase (delta 6)	*desD*	*sll0262*	-1.31	0.003173
membrane-associated rubredoxin, essential for photosystem I assembly	*rubA*	*slr2033*	-1.24	0.000295
Photosynthesis and respiration				
ATP synthase				
hypothetical protein	*atp1*	*sll1321*	-0.44	0.034410
ATP synthase A chain of CF(0)	*atpI*	*sll1322*	-0.62	0.000008
ATP synthase subunit b' of CF(0)	*atpG*	*sll1323*	-1.07	0.002997
ATP synthase B chain (subunit I) of CF(0)	*atpF*	*sll1324*	-1.03	0.004631
ATP synthase delta chain of CF(1)	*atpD*	*sll1325*	-1.14	0.005973
ATP synthase alpha chain	*atpA*	*sll1326*	-1.60	0.000283
ATP synthase gamma chain	*atpC*	*sll1327*	-1.47	0.001621
ATP synthase beta subunit	*atpB*	*slr1329*	-0.81	0.003780
ATP synthase epsilon chain of CF(1)	*atpE*	*slr1330*	-1.84	0.003305
ATP synthase C chain of CF(0)	*atpH*	*ssl2615*	-0.69	0.000249
CO_2 concentration mechanism				
CO_2 concentrating mechanism protein CcmK	*ccmK2*	*sll1028*	-1.21	0.000038
CO_2 concentrating mechanism protein CcmK	*ccmK1*	*sll1029*	-0.98	0.000255
CO_2 concentrating mechanism protein CcmL, putative carboxysome assembly protein	*ccmL*	*sll1030*	-0.80	0.000036
CO_2 concentrating mechanism protein CcmM, putative carboxysome structural protein	*ccmM*	*sll1031*	-2.04	0.001716
CO_2 concentrating mechanism protein CcmN, putative carboxysome assembly protein	*ccmN*	*sll1032*	-1.75	0.000308
CO_2 concentrating mechanism protein CcmO	*ccmO*	*slr0436*	0.58	0.006077
beta-type carbonic anhydrase localized in the carboxysome	*ccaA*	*slr1347*	0.16	0.070627
carboxysome formation protein CcmA	*ccmA*	*sll0934*	0.38	0.004437
CO_2 concentrating mechanism protein CcmK homolog 3, put. carboxysome assembly protein	*ccmK3*	*slr1838*	0.39	0.006325
CO_2 concentrating mechanism protein CcmK homolog 4, put. carboxysome assembly protein	*ccmK4*	*slr1839*	0.23	0.085332
Rubisco				
ribulose bisphosphate carboxylase large subunit	*rbcL*	*slr0009*	-1.41	0.000366
possible Rubisco chaperonin	*rbcX*	*slr0011*	-1.37	0.001161
ribulose bisphosphate carboxylase small subunit	*rbcS*	*slr0012*	-1.33	0.001451

Table S2. *continued*

Category and function	Gene	ORF	FC (log_2)	P-value
NDH-1 complex				
NADH dehydrogenase subunit 1	*ndhA*	*sll0519*	-1.89	0.000088
NADH dehydrogenase subunit 2	*ndhB*	*sll0223*	-2.02	0.000067
NADH dehydrogenase subunit 3	*ndhC*	*slr1279*	-1.42	0.001948
NADH dehydrogenase subunit 4 (involved in photosystem-1 cyclic electron flow)	*ndhD1*	*slr0331*	-0.02	0.894547
NADH dehydrogenase subunit 4	*ndhD2*	*slr1291*	-2.49	0.000026
NADH dehydrogenase subunit 4 (involved in low CO2-inducible, high affinity CO2 uptake)	*ndhD3*	*sll1733*	-1.91	0.001400
NADH dehydrogenase subunit 4 (involved in constitutive, low affinity CO2 uptake)	*ndhD4*	*sll0027*	-1.09	0.015006
NADH dehydrogenase subunit 4L	*ndhE*	*sll0522*	-1.61	0.000788
NADH dehydrogenase subunit 5	*ndhF1*	*slr0844*	-0.48	0.000178
NADH dehydrogenase subunit 5 (involved in low CO2-inducible, high affinity CO2 uptake)	*ndhF3*	*sll1732*	-2.96	0.000052
NADH dehydrogenase subunit 5 (involved in constitutive, low affinity CO2 uptake)	*ndhF4*	*sll0026*	-1.06	0.001344
NADH dehydrogenase subunit 6	*ndhG*	*sll0521*	-1.53	0.002975
NADH dehydrogenase subunit 7	*ndhH*	*slr0261*	-1.56	0.000116
NADH dehydrogenase subunit NdhI	*ndhI*	*sll0520*	-1.85	0.000051
NADH dehydrogenase subunit I	*ndhJ*	*slr1281*	-1.70	0.000286
NADH dehydrogenase subunit NdhK	*ndhK*	*slr1280*	-1.31	0.001896
NADH dehydrogenase subunit NdhL	*ndhL*	*ssr1386*	-0.36	0.194783
hypothetical protein	*ndhM*	*slr1623*	-1.32	0.001566
hypothetical protein	*ndhN*	*sll1262*	-1.05	0.002447
hypothetical protein	*ndhO*	*ssl1690*	0.71	0.007114
protein involved in low CO2-inducible, high affinity CO2 uptake	*cupA*	*sll1734*	-1.11	0.011503
protein involved in constitutive low affinity CO2 uptake	*cupB*	*slr1302*	0.27	0.022984
hypothetical protein	*cupS*	*sll1735*	-0.89	0.073693
mnh operon				
hypothetical protein	*mrpC*	*slr2006*	-3.36	0.000018
NADH dehydrogenase subunit 4	*ndhD5*	*slr2007*	-3.08	0.000155
hypothetical protein	*mrpC*	*slr2008*	-1.63	0.001452
NADH dehydrogenase subunit 4	*ndhD6*	*slr2009*	-1.33	0.011549
hypothetical protein	*mrpE*	*slr2010*	-1.65	0.003987
hypothetical protein	*mrpA*	*slr2011*	-1.64	0.001347
hypothetical protein	*mrpB*	*slr2012*	-1.27	0.003728
hypothetical protein		*slr2013*	-1.10	0.004208
hypothetical protein	*mrpF*	*ssr3409*	-1.48	0.001566
hypothetical protein	*mrpG*	*ssr3410*	-1.44	0.002697
sbt operon				
sodium-dependent bicarbonate transporter	*sbtA*	*slr1512*	-1.46	0.000101
periplasmic protein, function unknown	*sbtB*	*slr1513*	-2.14	0.038726
photosystem II PsbN protein	*psbN*	*smr0009*	-1.05	0.001000

Table S2. *continued*

Category and function	Gene	ORF	FC (log_2)	P-value
putative homolog of plant HCF136, which is essential for stability or assembly of photosystem II	*ycf48*	*slr2034*	-1.29	0.001703
phycobilisome core-membrane linker polypeptide	*apcE*	*slr0335*	-1.21	0.001033
iron-sulfur cluster binding protein homolog		*slr2059*	-1.07	0.003000
cytochrome c oxidase subunit III	*ctaEII*	*slr2083*	-1.02	0.002650
cytochrome c oxidase subunit I	*ctaDII*	*slr2082*	-1.27	0.000177
Purines, pyrimidines, nucleosides, and nucleotides				
nucleoside diphosphate kinase	*ndkR*	*sll1852*	-1.01	0.000359
Regulatory functions				
two-component hybrid sensor and regulator	*hik39*	*sll1296*	-1.25	0.000285
pleiotropic regulatory protein homolog	*degT*	*slr1666*	-1.04	0.001841
response regulator for energy transfer from phycobilisomes to photosystems	*rre26*	*slr0947*	-1.76	0.000039
Controlling expression of low carbon inducible genes				
ndhF3 operon transcriptional regulator, LysR family protein	*ccmR*	*sll1594*	-3.73	0.000131
cmp operon transcriptional regulator, LysR family protein	*cmpR*	*sll0030*	-0.89	0.058628
hypothetical protein		*sll0822*	-1.38	0.000284
DNA replication, restriction, modification, recombination, and repair				
similar to ultraviolet light resistance protein B	*umuC*	*slr0790*	-1.07	0.008127
putative modulator of DNA gyrase; TldD		*slr1322*	-1.71	0.000012
Transcription				
group2 RNA polymerase sigma factor SigD	*sigD*	*sll2012*	-1.57	0.000947
phosphoprotein substrate of icfG gene cluster	*icfG*	*slr1856*	-1.31	0.004378
RNA helicase Light	*crhR*	*slr0083*	-1.30	0.014926
principal RNA polymerase sigma factor SigA	*sigA*	*slr0653*	-1.19	0.000371
Translation				
probable pseudouridine synthase		*slr1592*	-2.55	0.000023
S-adenosylmethionine synthetase	*metX*	*sll0927*	-2.00	0.000061
aspartyl-tRNA synthetase	*aspS*	*slr1720*	-1.49	0.000216
RNA-binding protein	*rbp3*	*slr0193*	-1.14	0.002003
GTP-binding protein TypA/BipA homolog	*fus*	*slr1105*	-1.12	0.001271
30S ribosomal protein S10	*rps10*	*sll1101*	-1.01	0.009265
Transport and binding proteins				
ABC transporter ATP-binding protein		*sll0182*	-1.15	0.008599
ABC transporter ATP-binding protein		*sll0759*	-1.12	0.000622
ABC transporter permease protein		*sll1482*	-1.28	0.000267
ABC transporter, ATP-binding protein		*sll0778*	-1.33	0.000190
ATP-binding subunit of the ABC-type Nat permease for neutral amino acids	*natE*	*slr1881*	-1.21	0.006347
cation efflux system protein		*sll1263*	-1.53	0.001090
cation-transporting p-type ATPase PacL	*pacL*	*sll0672*	-1.28	0.000146
low affinity sulfate transporter	*bicA*	*sll0834*	-1.10	0.003168
membrane subunit of a Ktr-like ion transport system	*ktrB*	*slr1509*	-1.24	0.005553

Table S2. *continued*

Category and function	Gene	ORF	FC (log_2)	P-value
molybdate-binding periplasmic protein	*modA*	*sll0738*	-1.09	0.003616
Na+/H+ antiporter	*nhaS1*	*slr1727*	-1.16	0.001386
Na+/H+ antiporter	*nhaS3*	*sll0689*	-2.22	0.000059
Nitrate/nitrite transport system				
nitrate/nitrite transport system substrate-binding protein	*nrtA*	*sll1450*	-1.92	0.030050
nitrate/nitrite transport system permease protein	*nrtB*	*sll1451*	-1.88	0.042816
nitrate/nitrite transport system ATP-binding protein	*nrtC*	*sll1452*	-1.90	0.030542
nitrate/nitrite transport system ATP-binding protein	*nrtD*	*sll1453*	-2.17	0.006835
oligopeptide-binding protein of oligopeptide ABC transporter	*appA*	*sll1699*	-1.20	0.000247
potassium-transporting P-type ATPase B chain	*kdpB*	*slr1729*	-1.07	0.000435
RND multidrug efflux transporter	*envD*	*slr0369*	-1.03	0.000192
toxin secretion ABC transporter ATP-binding protein	*hlyB*	*sll1180*	-1.17	0.005413
water channel protein	*aqpZ*	*slr2057*	-1.83	0.000478
zinc transport system substrate-binding protein	*znuA*	*slr2043*	-1.08	0.008053
Other categories				
probable bacterioferritin comigratory protein		*sll1159*	-1.56	0.001092
DrgA protein homolog	*drgA*	*slr1719*	-1.24	0.000003
probable dioxygenase, Rieske iron-sulfur component	*pobA*	*sll1297*	-1.01	0.008807
probable hydrolase		*sll1305*	-1.15	0.010442
phenazine biosynthetic protein PhzF homolog		*slr1019*	-2.19	0.000011
flavoprotein	*flv2*	*sll0219*	-3.67	0.002601
flavoprotein	*flv4*	*sll0217*	-4.72	0.000179
flavoprotein	*flv3*	*sll0550*	-1.09	0.014806
periplasmic WD-repeat protein		*slr1410*	-1.49	0.008824
periplasmic WD-repeat protein		*slr1409*	-1.00	0.001603
Hypothetical				
hypothetical protein		*sll0141*	-2.01	0.000256
hypothetical protein		*sll0218*	-4.75	0.000535
hypothetical protein		*sll0350*	-1.29	0.003235
hypothetical protein		*sll0529*	-2.14	0.000060
hypothetical protein	*cruF*	*sll0814*	-1.44	0.000319
hypothetical protein		*sll0863*	-1.11	0.006420
hypothetical protein		*sll1071*	-1.01	0.000438
hypothetical protein		*sll1158*	-2.65	0.000053
putative C4-dicarboxylase binding protein, periplasmic protein	*dctP*	*sll1314*	-1.31	0.001100
hypothetical protein		*sll1526*	-1.59	0.000246
hypothetical protein		*sll1532*	-2.44	0.000219
hypothetical protein	*SynAco*	*sll1541*	-1.06	0.010479
hypothetical protein		*sll1660*	-1.12	0.008596
hypothetical protein YCF51	*ycf51*	*sll1702*	-1.04	0.025003
hypothetical protein		*sll2006*	-1.20	0.003371

Table S2. *continued*

Category and function	Gene	ORF	FC (log_2)	P-value
hypothetical protein		*sll2007*	-1.06	0.030662
hypothetical protein	*sufD*	*slr0076*	-1.65	0.010169
hypothetical protein		*slr0082*	-1.81	0.007744
hypothetical protein		*slr0105*	-1.00	0.016563
hypothetical protein		*slr0199*	-1.46	0.001653
hypothetical protein		*slr0263*	-1.26	0.012964
hypothetical protein		*slr0292*	-1.08	0.000279
hypothetical protein		*slr0320*	-1.74	0.000057
chaperon-like protein for quinone binding in photosystem II	*ycf39*	*slr0399*	-1.09	0.002351
hypothetical protein		*slr0400*	-1.04	0.000397
hypothetical protein	*rlpA*	*slr0423*	-1.35	0.012308
hypothetical protein		*slr0479*	-1.59	0.000076
hypothetical protein YCF46	*ycf46*	*slr0480*	-1.14	0.007983
hypothetical protein		*slr0732*	-1.43	0.000060
hypothetical protein		*slr0870*	-1.33	0.018744
hypothetical protein		*slr0941*	-1.05	0.000480
hypothetical protein		*slr0948*	-1.52	0.000629
hypothetical protein		*slr0964*	-1.25	0.015988
hypothetical protein		*slr1220*	-1.96	0.006203
hypothetical protein		*slr1263*	-1.08	0.001496
hypothetical protein	*pilO*	*slr1276*	-1.29	0.001112
hypothetical protein		*slr1342*	-1.06	0.000398
periplasmic protein, function unknown		*slr1406*	-1.02	0.001154
hypothetical protein		*slr1600*	-1.03	0.002727
hypothetical protein		*slr1753*	-1.14	0.002508
hypothetical protein		*slr1799*	-1.10	0.004538
hypothetical protein		*slr1906*	-1.32	0.007236
hypothetical protein		*slr1919*	-1.16	0.001407
hypothetical protein		*slr1990*	-1.19	0.000437
periplasmic protein, function unknown		*slr2005*	-1.29	0.000316
hypothetical protein		*ssl0352*	-1.09	0.001508
hypothetical protein		*ssl2874*	-2.51	0.000340
probable DNA-directed RNA polymerase omega subunit	*ycf61*	*ssl2982*	-1.79	0.000357
hypothetical protein		*ssr0550*	-1.08	0.009446
hypothetical protein		*ssr1473*	-1.01	0.011846
hypothetical protein		*ssr3588*	-1.29	0.000161
Unknown				
unknown protein		*sll0263*	-2.01	0.006770
unknown protein		*sll0775*	-1.24	0.020495
unknown protein		*sll1265*	-1.08	0.001219
periplasmic protein, function unknown		*sll1306*	-1.05	0.004715

Table S2. *continued*

Category and function	Gene	ORF	FC (log_2)	P-value
unknown protein		*sll1530*	-1.62	0.003415
unknown protein		*sll1531*	-1.94	0.000271
unknown protein		*sll1873*	-1.16	0.011595
unknown protein		*slr0106*	-2.64	0.002364
unknown protein		*slr0168*	-1.16	0.010677
unknown protein		*slr0262*	-1.19	0.000225
unknown protein		*slr0476*	-1.29	0.003250
unknown protein	*mvrA*	*slr0616*	-3.19	0.000013
unknown protein		*slr0871*	-1.81	0.005885
unknown protein		*slr1552*	-1.24	0.003320
unknown protein		*slr1636*	-1.72	0.000857
unknown protein		*slr1788*	-2.30	0.000019
unknown protein		*slr1855*	-1.35	0.001318
periplasmic protein, function unknown		*slr2004*	-1.22	0.000018
unknown protein		*slr2018*	-1.05	0.001807
unknown protein		*slr2046*	-1.33	0.000418
unknown protein		*ssl0410*	-1.95	0.000003
unknown protein		*ssl2814*	-2.53	0.001333

Table S3. Highly expressed genes in *Synechocystis* sp. PCC 6803 in standard growth conditions.

Category and function	Gene	ORF	$\log_2$ signal
Amino acid biosynthesis			
threonine synthase	thrC	sll1688	14.00
Biosynthesis of cofactors, prosthetic groups, and carriers			
heme oxygenase	ho1	sll1184	14.24
Cell envelope			
probable porin; major outer membrane protein		slr1908	15.38
probable porin; major outer membrane protein		slr1841	16.75
putative peptidase	nlpD	slr0993	15.45
Cellular processes			
cell division protein FtsH	ftsH	slr1604	14.28
10kD chaperonin	groES	slr2075	14.50
60kD chaperonin	groEL1	slr2076	15.04
Energy metabolism			
adenosylhomocysteinase	ahcY	sll1234	14.22
pentose-5-phosphate-3-epimerase	cfxE	sll0807	14.25
transketolase	tktA	sll1070	14.39
fructose-bisphosphate aldolase, class II	fbaA, fda	sll0018	15.36
Fatty acid, phospholipid and sterol metabolism			
acyl-lipid desaturase (delta 12)	desA	slr1350	15.13
Photosynthesis and respiration			
allophycocyanin alpha subunit	apcA	slr2067	17.45
allophycocyanin beta subunit	apcB	slr1986	16.98
apocytochrome f, component of cytochrome b6/f complex	petA	sll1317	14.00
ATP synthase A chain of CF(0)	atpI	sll1322	15.46
ATP synthase alpha chain	atpA	sll1326	14.19
ATP synthase beta subunit	atpB	slr1329	14.52
ATP synthase C chain of CF(0)	atpH	ssl2615	15.07
ATP synthase delta chain of CF(1)	atpD	sll1325	14.00
carbon dioxide concentrating mechanism protein CcmK	ccmK2	sll1028	14.11
carbon dioxide concentrating mechanism protein CcmK	ccmK1	sll1029	14.69
carbon dioxide concentrating mechanism protein CcmL	ccmL	sll1030	14.30
cytochrome b559 alpha subunit	psbE	ssr3451	14.18
cytochrome b6	petB	slr0342	15.89
cytochrome b6-f complex iron-sulfur subunit (Rieske iron sulfur protein)	petC1	sll1316	14.00
cytochrome b6-f complex subunit 4	petD	slr0343	15.32
ferredoxin I, essential for growth	petF	ssl0020	15.53
hypothetical protein	atp1	sll1321	14.31
NADH dehydrogenase subunit 1	ndhA	sll0519	15.08
NADH dehydrogenase subunit 2	ndhB	sll0223	14.41
NADH dehydrogenase subunit 3	ndhC	slr1279	15.19

Table S3. *continued*

Category and function	Gene	ORF	log_2 signal
NADH dehydrogenase subunit 4	ndhD2	slr1291	15.36
NADH dehydrogenase subunit 4	ndhD3	sll1733	15.16
NADH dehydrogenase subunit 5	ndhF3	sll1732	15.09
NADH dehydrogenase subunit 6	ndhG	sll0521	14.55
NADH dehydrogenase subunit NdhI	ndhI	sll0520	14.12
NADH dehydrogenase subunit NdhK	ndhK	slr1280	15.35
P700 apoprotein subunit Ia	psaA	slr1834	18.10
P700 apoprotein subunit Ib	psaB	slr1835	17.70
photosystem I reaction center subunit III precursor (PSI-F)	psaF	sll0819	16.31
photosystem I subunit II	psaD	slr0737	15.25
photosystem I subunit IX	psaJ	sml0008	15.79
photosystem I subunit VII	psaC	ssl0563	14.22
photosystem I subunit XI	psaL	slr1655	15.30
photosystem II CP47 protein	psbB	slr0906	15.33
photosystem II CP43 protein	psbC	sll0851	16.39
photosystem II D1 protein	psbA3	sll1867	18.10
photosystem II D1 protein	psbA2	slr1311	18.13
photosystem II D2 protein	psbD2	slr0927	16.86
photosystem II D2 protein	psbD	sll0849	17.14
phycobilisome core component	apcF	slr1459	14.22
phycobilisome core-membrane linker polypeptide	apcE	slr0335	14.59
phycobilisome rod linker polypeptide	cpcC1	sll1580	15.38
phycobilisome rod linker polypeptide	cpcC2	sll1579	15.95
phycobilisome rod-core linker polypeptide	cpcG1	slr2051	15.30
phycobilisome small rod linker polypeptide	cpcD	ssl3093	14.19
phycocyanin alpha subunit	cpcA	sll1578	18.17
phycocyanin beta subunit	cpcB	sll1577	17.95
Plastocyanin	petE	sll0199	16.41
ribulose bisphosphate carboxylase large subunit	rbcL	slr0009	16.65
ribulose bisphosphate carboxylase small subunit	rbcS	slr0012	14.99
DNA replication, restriction, modification, recombination, and repair			
putative endonuclease [encoded in trnfM-intron: 2791054 - 2791708]	trnfM	slr0915	15.45
Transcription			
RNA polymerase alpha subunit	rpoA	sll1818	14.12
principal RNA polymerase sigma factor SigA	sigA	slr0653	14.29
Translation			
30S ribosomal protein S1	rps1a	slr1356	14.25
30S ribosomal protein S3	rps3	sll1804	14.97
30S ribosomal protein S5	rps5	sll1812	14.17
30S ribosomal protein S7	rps7	sll1097	14.54
30S ribosomal protein S8	rps8	sll1809	14.35

Table S3. *continued*

Category and function	Gene	ORF	log_2 signal
30S ribosomal protein S9	rps9	sll1822	14.84
30S ribosomal protein S11	rps11	sll1817	14.35
30S ribosomal protein S12	rps12	sll1096	14.86
30S ribosomal protein S13	rps13	sll1816	14.18
30S ribosomal protein S15	rps15	ssl1784	14.14
30S ribosomal protein S16	rps16	ssr0482	14.72
30S ribosomal protein S17	rps17	ssl3437	14.53
30S ribosomal protein S19	rps19	ssl3432	15.29
30S ribosomal protein S20	rps20	ssl2233	14.08
50S ribosomal protein L1	rpl1	sll1744	14.12
50S ribosomal protein L2	rpl2	sll1802	14.74
50S ribosomal protein L3	rpl3	sll1799	14.70
50S ribosomal protein L4	rpl4	sll1800	15.43
50S ribosomal protein L5	rpl5	sll1808	14.27
50S ribosomal protein L6	rpl6	sll1810	14.45
50S ribosomal protein L10	rpl10	sll1745	14.80
50S ribosomal protein L12	rpl12	sll1746	14.11
50S ribosomal protein L13	rpl13	sll1821	14.61
50S ribosomal protein L14	rpl14	sll1806	14.48
50S ribosomal protein L15	rpl15	sll1813	14.38
50S ribosomal protein L16	rpl16	sll1805	15.41
50S ribosomal protein L18	rpl18	sll1811	14.70
50S ribosomal protein L20	rpl20	sll0767	15.30
50S ribosomal protein L21	rplU	slr1678	14.94
50S ribosomal protein L22	rpl22	sll1803	15.19
50S ribosomal protein L23	rpl23	sll1801	15.20
50S ribosomal protein L24	rpl24	sll1807	14.81
50S ribosomal protein L27	rpl27	ssr2799	14.56
50S ribosomal protein L29	rpl29	ssl3436	15.10
50S ribosomal protein L33	rpl33	ssr1398	14.36
50S ribosomal protein L35	rpl35	ssl1426	15.00
50S ribosomal protein L36	rpl36	sml0006	14.07
ATP-dependent Clp protease ATPase subunit	clpC	sll0020	14.47
ATP-dependent Clp protease proteolytic subunit	clpP4	slr0164	14.92
elongation factor Tu	tufA	sll1099	15.57
tRNA pseudouridine synthase 1	truA	sll1820	14.25
Other categories			
antioxidant protein	1-cys prx	slr1198	14.08
Flavodiiron protein	flv3	sll0550	14.54
Hypothetical			
glutamine synthetase inactivating factor IF7	gifA	ssl1911	14.77

Table S3. *continued*

Category and function	Gene	ORF	log_2 signal
hypothetical protein		ssl0483	14.10
hypothetical protein		sll1832	14.24
hypothetical protein		slr0287	14.45
hypothetical protein		slr0376	14.92
hypothetical protein		slr1634	15.34
hypothetical protein		slr0374	15.75
hypothetical protein		slr0373	15.91
hypothetical protein YCF59	ycf59	sll1214	14.66
periplasmic protein, function unknown	sbtB	slr1513	15.23
possible Rubisco chaperonin	rbcX	slr0011	16.33
protein involved in low CO2-inducible, high affinity CO2 uptake	cupA	sll1734	15.26
sodium-dependent bicarbonate transporter	sbtA	slr1512	16.88
water-soluble carotenoid protein	ocp	slr1963	15.14
Unknown			
unknown protein		slr0476	14.90
unknown protein		sll1338	15.39
glutamine synthetase inactivating factor IF17	gifB	sll1515	15.41
unknown protein	HlyA, hlp	sll1951	16.16

Signal intensities >14.00 (log_2) from DNA microarray were used as a threshold value for highly expressed genes.

Table S4. Information-content-maximizing alignments of promoter regions of genes up-regulated or down-regulated in ΔrpoZ.

Gene name	Distance	Alignment
Down-regulated genes		
sll0807	16	ttttaaaggccgggtttgggggcctACTACAattgaccgtaattgttTACACTgaatttc
sll1071	17	ttgtccagaaaagatcagccggtaTGAAAAtttaacggaggcaatacCTTAAAataaggt
slr2005	18	ttggggattaaattttgtccctgcCCCAAAgcccaaaaccattgttgtTATTATgctggg
slr1843	17	ttcctgaaaactttgccagaaaaagAAAACTttcgggaattgtgagttATTTTAaagcgt
sll0726	17	ttatctctttgcccatggatagcccTTGGCTagttaacccttttggcTAGGATttgggt
sll1262	17	tgtgtcaaaatcgtgggtaacttaGTCTGTcctaggcttccataactTATAATtatcgcc
slr0082	18	tgtataacattacaccaaacgaccTCAGGTtttctggtgtaaatctgtTACAATgtcttt
slr2033	16	tgatgaatccccattgagaaggctgGGGAAAagggtagcgaagatgtCATAATttttaac
sll0370	16	tcaataaacctggatatgggaacgggTGGCGAtcgccgataaagcctaTACAATgattgg
slr1788	18	tattatttaaatatctaccaaaggGATGATggcagaagaatttggtaTATAATgccgcc
sll0834	18	tatagattttatctatctgtatcaTTGCCTgaagtaaaaaccagccctAGAATAttctct
slr1513	16	tagtttatggtctgtcaataatccctTGACCTaacagaccaaaaagatTAACTTacatac
slr1220	18	taaataagcattcctagcaatttTGCCCAagaaaatcaagccattcgCTACAAtgttttc
slr1990	16	gggccaaaccatagggagttttcccTAGAAAgcctagaagtttttggATATAAtaagcaa
slr0839	16	gggacgccgtttgtaggttgggattcTTTCCTtttcgccatctattgtTACACTtggtaa
sll0263	16	ggctagaggccatgggcaaagcatcgTGACATtgccttgggattgaagCAAAATggcaaa
slr1330	18	ggagacattgaagaagctaaggccAAAGGTgccaagctcaaagagggtTAAGTTaataac
slr1277	17	gctggtagattatcgctaacggtctTACGATttttgaataattattcaATAATTgatttt
slr0476	16	gagggaaaagaggtcagaagtcatggcGGCAAAggagctatagtctgggATAATAgggct
slr2008	16	gactctggggtttagccatcccattgGGCAAAgaacgatggcatttgaTAACCTggggag
sll1526	16	gaaggaaaatttgtgccattcaactaGGCCCCaaagcccctttcttgtAAGCTAagggct
sll0529	17	ctttttaatggcagaatagggggaTTATCTggtgttccccaaggggggTAAAATtagcctc
sll1234	18	ctgtcaatggacaaaggggcgatcGCCATTccagaccgataaaatgttAAGTTAtatcac
slr0335	17	ctcctacataaaagttaacaaagcCCCTGTagcgttccgaaacgttcTACCCTagatgct
sll1212	17	ctcaccatggcgatcgccagaaggcCTGCATctgggcaaggattctgaTATTTTtagggg
ssl0352	18	ctatggttaactctaggtttaggGGCGAAagaaaacctgctcctttgCTAGAAttgtcca
slr1516	18	cggttctccaccggattaccgaccTTTTAAccgaggaaactttagggtAAAGTGagagcc
slr1350	17	cgcggctgaattcagaagcaattcgGTTCCTggtcaatggcaacgtgtTATAAAaagaaa
sll1702	16	ccctgcggcgatcggggggatgaagaTTAACTaactgcattttctgttAGATTTgttgac
ssl0410	17	ccctccagcaaacaggagaaactccGGCGATcgccatttcccatgattTACCCTaggggt
sll1688	17	cccgataaaattagtcgggattaatTGACGCccatttgccccgtgttAGCATGccgaaa
slr1666	17	catggatggtgggcggctaatctgtTCAACTaagggacggccatgcccTAGAATttgatg
slr1279	17	cataggttaaacatatagttcagggCTTGGTctgtcctggttggagtcCTATAAtcagaa
slr2006	17	caggggattttatggcgatcgccatTGCTATtggtaccaaaacaaggaTAGAATctatgc
sll0018	16	caggacaaaaaagatcgttaaaaatTGTCAAggcgcagaaactagccTAAGATgagaccc
slr1799	17	cagcctagcgccatctctgggttaaAATCCTctcaggtcaagtaatgtTAAGATtggcca
sll1873	18	caaatcggggctgactggccataGGAAATtccagcgccctattttgTTAAAAttcagtc
slr0076	16	attttccattctgctaggcaagttgGCCGATcgccaggcattattgaGACAATttgagct
sll0822	17	attaactatttatttgaacaaactaGATAATtattccaagaaacgtgtAATATAagcagg
slr0199	17	atggggtaggtgggtgaggaagtTATTATctagaggtgtgtgaggaGTAAGTtgaagccc
sll1852	18	atcgatggggctggttaccattCATAATcccgatgcccaaaccatgTAAAATacagggt
slr2082	17	atccagaacaaatttaggaacaatggGGCCCAcggcatggctttctgttAAAAATaaaca
sll0814	18	atcaattaacccaccgctcctggtAAAGATttgtaaactataatgactTAGAATttgaat
smr0009	16	atagacttcccgtaaaaatgactaaATGAGTttttgtaatgttctgtAATAATataagac
sll0223	18	ataaattccatcacccattctaatTCTCCTttgcggcaaacctgcgctAAGCTTattcgt
slr1105	17	aggttcagttttagaagctttttgcTCCAGAggaggcaacggtaggatAGACTGaatatt
slr0292	17	agctctgaatttggttaaaatcgtcACCCCAgggtagggatgtgttgaTTAAGAtatatt
slr1906	18	agcaaactgcaaatggagcccagTCTCCTcggagtaaaaaatttttGTATAAttccggg
sll0026	17	agaccgacaacggcgatcgccaaagTCAGAAttgaggcagcgatagcgTAAAATctgatc
slr1727	17	actgacccggacccaaaggtttatttTGCCCTcagagcggagtggccccATAATGgggga
slr0193	17	accggggcggacgggactgtccccaTGGCCTggtgtctattatcgtcaTAAACTtggtta
sll0262	16	aatttttagtctcccccggcgctggaGTTTTTttgtagttaatggcggTATAATgtgaaa
slr1322	17	aatatctactgtcttctcggctagaGTCAAAccttacactgagttggtTAGAATacatta
slr0399	16	aacaaattttttgtgccctggccccaaTACCCAtaattgtcagagtctgATAGGAtttagc
slr0732	18	aaataattatcattgcaaactgggCGAAATttggctgatcattgccgaTAAAATatggcg
Up-regulated genes		
sll0771	18	ttttgcaacatttaattaagaatTCATGAcgaagttttttacttggTAGGATttattcc
sll1583	18	ttgcgactatttcgccccgtcgtCCCATAaaaggttcctaaacttgcTATAAGaggatct
sll1247	16	ttcatgggaaagaataggagtgaaaaATATGGcggaggaaaactgagtTAGAATtgactc
ssr0693	17	ttatgttaactatcttaaaatactaGATAGAaaaatttaagtagcggaTATAATtatttt
slr0226	18	tggcgatcgccgtcataaaaattaGCTTGCtatcccttcaatgtagatGATAATgggaat
ssr2153	16	tgccactgcagaaaaaatcgagcatTCTGGAttatgaggattacggtTAAAATagttggc
slr0244	17	tgagaaagtccgcaacaagtcgttaAATTTCtgcgcttgcattggcctTATTCTgaaggg
slr0144	18	tgacaacgttttgtaacagaggtTTCTCCcgcctacaaaagctatgtAAGAATtagggtt
slr1689	17	tgaattggaattggtgcaagcggcgGCAAAAtagtcttgactgtggacTAAATTtgcatt

slr0888	17	tctgtaaagatccatctcaattttTCCTCGgaactaaatcccctgccAATAATgaaggt
sll0038	17	tcgatgaagttttcttgcgtactttGGCGACccataccccttcagggCAAGCTatagat
slr1811	18	tcacatccggctcaagatgccaatTTTGGGgacaattttgctaaggcTACAATgaagct
slr2107	17	tcaagtaacggtttgttacgggtgTTAAGGgttgggggtttgttggaTAAGCTgatgagt
ssl2384	16	tagttgcaaaaaccgccctttctctCCCTTTtcccccgggggagtgaTACGTTgtaaaac
slr1667	16	tagtgaaagtccagtattagtgccaaTACGCAcaaattgtcatttcccTACAATaaagaa
sll1236	16	gttggaccacacagggtcgacaagttTGAGTTaataatcagcaagcctTAACATtatttt
slr1584	18	gttacatttcgcaacatgaattaaCCCAGAcctgttcaccccttcaggTAAGCTgaggtt
slr0288	18	gtcgttgtttttgtatctatattgTCTATTttaaaaaatcatcttgcgTATGATtggggg
slr0889	18	ggggaaatgccggcggccatgatgcCCTTGCactggctctattggggacTAGAATtagtt
sll0335	16	ggcaaaacactttcactctgcaaataGCCAAAaactgatcaccaatggCACCATggccca
slr1958	16	gctgtaacagcggggcgtttagagatGGAAGAaatttaaaaaatttgaGAAGATtaatcc
slr0112	17	gatcgccaaaatggccctggacaaTGAAAAtttcccttgccatgggcTAGGATcgattgc
sll0022	17	ctttcgtaaataatttttaccaattAGTTTAttccgagaaaatctcaaTACAATaattcc
slr2052	17	cttctaacaattatggaacctctgCCCATAatcccagactaaaaaccTATACTtgccccc
slr1768	17	cttcggtattagcactattttctggGCAAACaattgaaaaaaataagcTAGCATggcttc
sll0198	16	ctcgccccgggaggatcaccgcaaaATATTCaccataacgaatttgcTAGTTTgaaaggt
slr0772	18	ctcgatcaaaatgtaaagttactgCTCGACaaggttaaggcttttaccGACAATttgagg
slr2098	16	cggcgacgctgttcgtcattattgggGCAGGAataagactaggtggatTAGCATagtcat
ssl2250	16	cctacggcaaaccggccccaaaaaatTGTTTTttctgccctgttgatgCATAATgaaaat
sll1330	17	ccccccatgcaggtaactgttgttaCAAAGCcttgacattgactttgtTAGATTaacagg
sll1514	16	ccccaccttctttcctgaaaaccgaaTCTAACctggaaggggaaatttTAAGATagaacc
sll1196	16	caattttaagaattctcacaaagaagTTTTGCtgattcgatttgcttcTACAAAtaaaac
slr0967	16	caaggaaattagcccgtttgggtgaaGACAGCttgggtagtctcccttTATCGTgagatc
slr0301	18	atttataaataattaccaacaaaaTAAAGTttactatatcaacctcgaTAAACTgaaggt
slr2108	17	atgggtaacggtttgttacgggtgTTAGGGgttggggcttggttgggTATGCTggtggct
slr1813	18	atcgccagataatgaggcaaatcgTGTGCCcactcattgagtggaagcTAAAATaagatt
ssr2078	17	atccctgggtttctgattatggtgTTTAGAgcccatttataaagtctTATAATggatagc
slr0217	16	atcattaagttgttgtaaacaaataTCAGTCcccactcaggaatcgtTAACATggaggcc
sll1426	17	agttgcagaaatagcaccctttttgcCCCAGCtttttagggaaaaatgcTATGGTcaatgt
sll1356	18	agtcatacaaaagttagcaatgGTAACCaaattttaattctggtccGACACTataaggga
sll0572	18	aggcaaagtattgtaacaaaggtTCTGGGaataatcccgttgctgttTATGATaaaaggt
ssl0832	18	agcggcctacatagttaattctcggGGTTTCcctaccaaaatcttgagtTAAGCTggaag
slr0749	18	agccagattaataccccaaactatTTCTCCaagaccccaaacctcgttAATAATgcgatc
sll0542	18	agaaatacaaaaggttacaaatcTGGGGAaaggggaaaaattttgcGACAATcatcaac
sll1502	16	acttaatagattcattaaggaaacctACCTCCcccccgtaatgaccgcTACAATtcaaaa
slr1928	16	acgtgtttatgctgaatgtgtcctggGTCACTcaatggtgaatggtgtTAGAGTaatatc
slr0148	17	acggctcaaaccagtttctgcttggTTAACAgaaggccatgaccatgcCACCATtatgga
slr1295	18	acctaattaaataatcagaaaatTACTCAttatatctccactgctgaTAAAATtattgag
sll1764	16	accgtcgttggcctgatgggggcggcTTCATCaaccgttggtaaggctTATCATtgagat
ssl3573	16	acaggtttacggcgatattcggtcgatTTTGGAaaacttgtctttaattTAAAATgtaaa
slr1957	17	acaaagttaagttttatggcattttTCTTTCctcccaaatagccttgcTAAGATagcggg
ssl1263	17	aatttttgctacaatttatgccagtTTCTTAgctagttgggccaaaacTAGAATaacttc
slr0680	17	aatttgaagaaagcttaacaatcctAACGGCgatcgactcttccccccCACAATagaaac
sll1286	17	aattatgaagatttctgttgaccagTGAGGCgatcgggattttcccgCACAATaacagt
ssr1375	16	aaatgttaaagactgttgcagaagtgTGCGTCtccgttaatgccatggTAGTTTttatag
sll1882	16	aaatccttacgttgttaataaaaagcgGGATTAatttcattctttaaacCATCATtaaat
slr0623	16	aaaagatgaataaattctaagtttcacGATATTtttccatacaggggtcAACAATtggtt

The sequence alignment is shown according to the -10 element, and the Distance column shows the number of nucleotides between the -10 and the -35 element.

The list of down-regulated genes shows alignments of promoter regions of genes whose FC(log_2) is -1.0 or lower in ΔrpoZ (information content of -10 region: 1.039 bits; -35 region: 0.239 bits; number of sequences: 55).

The list of up-regulated genes shows alignments of promoter regions of genes whose FC(log_2) is +1.0 or higher in ΔrpoZ (information content of -10 region: 0.884 bits; -35 region: 0.203 bits; number of sequences: 57).

SUPPLEMENTAL REFERENCES

1. Boratyn,G.M, Schäffer,A.A., Agarwala,R., Altschul,S.F., Lipman,D.J., Madden,T.L. (2012) Domain enhanced lookup time accelerated BLAST. Biol Direct 7:12.

2. Kaneko,T., Sato,S., Kotani,H., Tanaka,A., Asamizu,E., Nakamura,Y., Miyajima,N., Hirosawa,M.,Sugiura,M., Sasamoyo,S. et al. (1996) Sequence analysis of the genome of the unicellular cyanoacterium *Synechocystis* sp. strain PCC6803. II. Sequence determination of the entire genome and assignment of potential protein-coding regions. DNA Res 3: 109-136.

3. Kanesaki,Y., Shiwa,Y., Tajima,N., Suzuki,M., Watanabe,S., Sato,N., Ikeuchi,M. and Yoshikawa,H. (2012) Identification of substrain-specific mutations by massively parallel whole-genome resequencing of *Synechocystis* sp. PCC 6803. DNA Res. 19: 67-79.

4. Trautmann,D., Voss,B., Wilde, A., Al-Babili,S. and Hess,W.R. (2012) Microevolution in cyanobacteria: re-sequencing a motile substrain of *Synechocystis* sp. PCC 6803. DNA Res 19: 435-448.
